# Tecovirimat-Related Substance: Characterization, Hirshfeld Analysis, Theoretical Study, In Silico Toxicity Assessment

**DOI:** 10.3390/molecules31030502

**Published:** 2026-01-31

**Authors:** Fengfeng Wang, Xiaowen Hu, Caiyu Zhang, Lin Luan, Zhengzheng Zhou, Yang Liu

**Affiliations:** 1 Institute for Chemical Drug Control, National Institutes for Food and Drug Control, Beijing 102629, China; wangff@nifdc.org.cn (F.W.); huxiaowen@nifdc.org.cn (X.H.); zhangcy@nifdc.org.cn (C.Z.); luanlin@nifdc.org.cn (L.L.); 2 NMPA Key Laboratory for Safety Evaluation of Cosmetics, Department of Hygiene Inspection & Quarantine Science, School of Public Health, Southern Medical University, Guangzhou 510515, China; 3 Guangdong Provincial Key Laboratory of Tropical Disease Research, Department of Hygiene Inspection & Quarantine Science, School of Public Health, Southern Medical University, Guangzhou 510515, China

**Keywords:** tecovirimat, related substance, SCXRD, Hirshfeld analysis, in silico toxicity

## Abstract

Crystal structures of 4-(trifluoromethyl)benzene-1-carbohydrazide (**1**), 4-(trifluoromethyl)-N’-{[4-(trifluoromethyl)phenyl]carbonyl}benzohydrazide (**2**) and (1*R*,2*S*,6*R*,7*S*,8*S*,10*R*)-4-({[4-(trifluoromethyl)phenyl]carbonyl}amino)-4-azatetracyclo [5.3.2.0^8,10^.0^2,6^]dodec-11-ene-3,5-dione (**3**) were first reported. Besides the three new single-crystal structures, the single-crystal structure of tecovirimat (**4**) was also described herein. Hirshfeld analysis quantified intermolecular interactions, and PXRD confirmed high crystal phase purity. DSC data and packing energy calculations demonstrated that the melting point order matched the magnitude of packing energies for compounds with similar structures and analogous intermolecular interactions. According to the ICH M7 guidelines, in silico genotoxicity assessment using Derek Nexus and Sarah Nexus indicated that **2** showed no structural alerts for genotoxicity (ICH M7 Class 5), whereas **1** and **3** were classified as ICH M7 Class 3 due to structural features associated with potential genotoxicity, warranting further experimental evaluation.

## 1. Introduction

The World Health Organization officially declared smallpox—an infectious disease characterized by high contagiosity and potential fatality—eradicated in 1980. However, countries around the world still continue to conduct research on drugs for smallpox treatment [1]. Tecovirimat, an antiviral agent, was first identified through a high-throughput screening campaign in 2002. It exhibits broad-spectrum efficacy against all members of the *Orthopoxvirus* genus, including vaccinia virus, cowpox virus, ectromelia virus, rabbitpox virus, monkeypox virus, and Variola virus (the causative agent of smallpox) [2,3]. Tpoxx (tecovirimat) capsules were originally approved in 2018 to treat human smallpox disease in adults and pediatric patients [4]. In 2022, an intravenous formulation was approved for the same use. Also in 2022, the pediatric population was expanded to include patients weighing at least 3 kg [5]. Subsequently, Health Canada granted its approval in December 2021 [6], with the European Commission following suit in January 2022 [7]. In January 2025, SIGA Technologies announced that its antiviral TEPOXX has won regulatory approval in Japan for smallpox, mpox, and cowpox treatment [8].

Tecovirimat’s mechanism of action [9,10], elucidated via structural biology, targets orthopoxvirus-encoded phospholipase F13 (VP37)—a key factor in viral envelope formation and extracellular release. It binds to the F13 homodimer interface cavity, stabilizing the dimer via polar/hydrophobic interactions (EC_50_ = 92 nM), which disrupts F13’s binding to host Rab9 GTPase and TIP47. This blocks viral envelope assembly, inhibiting viral particle release and in vivo spread.

**Scheme 1:**
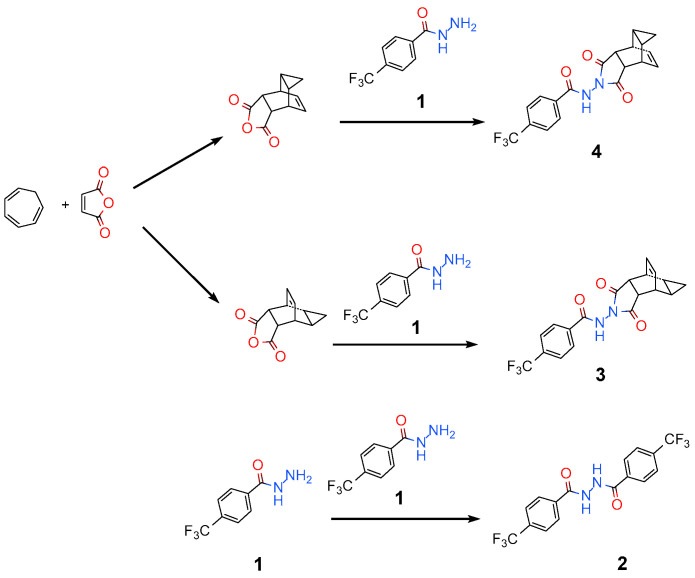
Synthesis process of 4 and its process-related impurities. Oxygen and nitrogen are colored red and blue, respectively, solely for clarity and ease of distinction.

A common synthetic route [11,12] of tecovirimat (**4**) is shown in Scheme 1 below. First, with cycloheptatriene and maleic anhydride as starting materials, the key intermediate is generated through a high-temperature pericyclic cascade process (6π-electrocyclisation and Diels–Alder cycloaddition). Then, the aforementioned intermediate undergoes a condensation reaction with a commercially available acyl hydrazide in ethanol to obtain the target drug. After optimization, the yield of this reaction is above 80% in each step [13].

Although the yield of each step in this process exceeds 80%, a significant amount of process-related substance remains in the ethanol solvent in the final reaction mixture. During the continuous evaporation of ethanol, multiple process-related substances may co-precipitate [14,15]. Notably, the single-crystal structures of these corresponding process-related substances have never been reported. Single-crystal X-ray diffraction (SCXRD) technology enables the precise structural elucidation of impurities or polymorphs [16,17], serving as a core step in ensuring product quality and driving process optimization across pharmaceutical development and materials synthesis. Building on this foundation, SCXRD not only allows the retrospective deduction of impurity formation pathways and the revelation of side reaction mechanisms during synthesis [18] but also provides critical structural insights for impurity toxicity assessment [19,20], particularly through the quantitative structure–activity relationship prediction (QSAR) of genotoxic impurities. Beyond the aforementioned applications, it also contributes significantly to the development of separation and detection methods [21].

In this study, three representative process-related substances were selected for single-crystal structure determination. The three related substances are 4-(trifluoromethyl)benzene-1-carbohydrazide,4-(trifluoromethyl)-N′-{[4-(trifluoromethyl)phenyl]carbonyl}benzohydrazide, and (1R,2S,6R,7S,8S,10R)-4-({[4-(trifluoromethyl)phenyl]carbonyl}amino)-4-azatetracyclo [5.3.2.0^8,10^.0^2,6^]dodec-11-ene-3,5-dione, which are designated as **1**, **2**, and **3**, respectively. The substance **1** serves as one of the starting materials for this reaction. **2** is a product derived from the dimerization reaction of **1**. The substance **3** is the product obtained from the reaction between **1** and the by-product generated in the previous Diels-Alder reaction. Herein, the single-crystal structures of these compounds were reported for the first time. The reactivity and electronic properties of these compounds were elucidated on the basis of electrostatic potential (ESP), highest occupied molecular orbital (HOMO) and lowest unoccupied molecular orbital (LUMO) analyses. Their hydrogen bond motifs were compared to gain insights into their packing modes, and Hirshfeld surface analysis was further performed to deepen the understanding of such packing behaviors. In addition to single-crystal X-ray diffraction, Fourier transform infrared (FTIR) spectroscopy, powder X-ray diffraction (PXRD), and differential scanning calorimetry (DSC) were also employed for characterization. The differences in melting points were rationalized by interaction and lattice energy calculations. Finally, considering that these compounds contain the hydrazide group, in silico toxicity predictions were conducted for these process-related substances.

## 2. Results and Discussion

### 2.1. Crystal Structure Analysis

**Figure 1:**
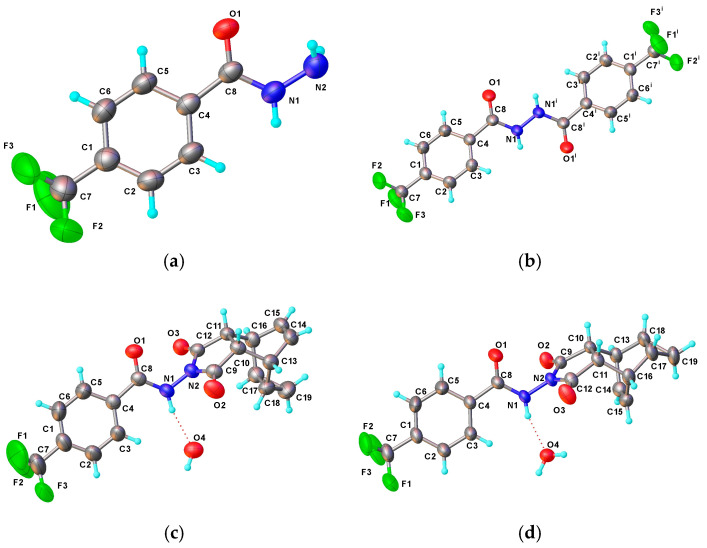
Ellipsoid diagram for **1** (**a**), **2** (**b**), **3** (**c**), and **4** (**d**) with 50% probability thermal ellipsoids, disorder part was omitted for clarity. The color code for atoms is defined as follows: gray for carbon, blue for nitrogen, red for oxygen, green for fluorine, and cyan for hydrogen.

From the perspective of the compounds’ structures, **2** is the dimer of **1**, and **3** is the epimer of **4**. Despite similarities in their structures, these four compounds crystallize in completely different space groups with total different cell parameters. The corresponding molecular ellipsoid diagram is presented in Figure 1. Table 1 lists the main crystallographic data for **1**–**4**. The crystal structures of **1**–**3** are reported for the first time. Although the crystal structure of **4** has been previously deposited in Cambridge Structural Database [22,23] (CSD Entry: UPUDOZ), the publicly accessible CIF (Crystallographic Information File) does not contain complete diffraction data, which limits the verification of structural reproducibility [24]. In the present study, we re-determined the room-temperature crystal structure of **4** and supplemented it with complete and traceable diffraction data.

All compounds feature functional groups such as hydrazide and a benzene ring, with their bond lengths falling within the typical range. In the present study, the bond lengths of these groups are also very close, and the corresponding data are shown in Table 2 below. In the hydrazide groups, the carbonyl and hydrazine moieties are coplanar. The dihedral angles between the benzene rings and the hydrazide planes differ among the four compounds. The dihedral angle data can be illustrated by the torsion angle information of C3–C4–C8–N1 with the corresponding data presented in Table 2 below. From the data in the table, it can be seen that **3** and **4** are similar in structure, with their torsion angles being very close to each other. The torsion angle of **2** is much larger than those of the other three compounds, and this difference arises from the fact that **2** undergoes distortion to form intermolecular hydrogen bonds.

**Figure 2:**
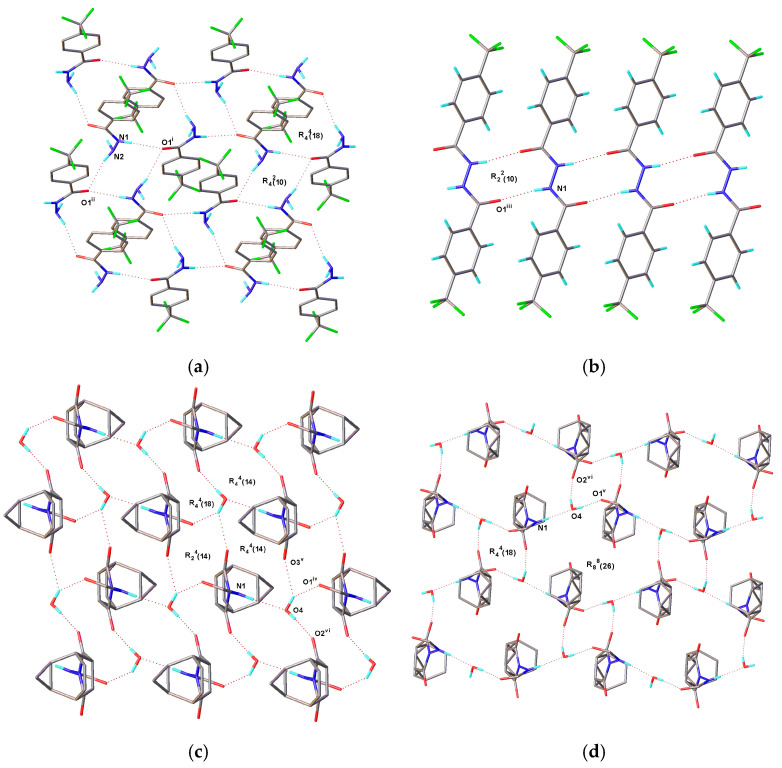
Ellipsoid diagram for **1** (**a**), **2** (**b**), **3** (**c**), and **4** (**d**) with 50% probability thermal ellipsoids, disorder part was omitted for clarity, trifluorotoluene was not shown in (**c**,**d**) for clarity. The red dashed lines represent hydrogen bonds. Symmetry codes: i: 1/2+X,1/2−Y,1−Z; ii: 3/2−X,1/2+Y,+Z; iii: +X,1+Y,+Z; iv: −1+X,+Y,+Z; v: 1−X,1−Y,1−Z; vi: 1−X,2−Y,1−Z.

The substance **1** crystallizes in the *Pbca* space group, with one molecule contained in each asymmetric unit. The crystal packing of **1** is dominated by robust intermolecular N1-H1...O1 (1/2+X, 1/2−Y, 1−Z) hydrogen bonds, which give rise to the formation of infinite chains along the x-axis. Additionally, intermolecular N2–H2A...O1 (3/2−X, 1/2+Y, +Z) hydrogen bonds induce the assembly of infinite chains along the y-axis. This y-axis hydrogen-bonded chain, together with the aforementioned x-axis chain, further constructs a continuous hydrogen-bonded network. These novel crystals show the diversity of H-bonding motifs using Etter [25] and Grell’s [26] graph set notation. Based on Etter and Grell’s methods for hydrogen bonds, *R*_4_^2^(10) and *R*_4_^4^(18) motifs can be observed in this network. The corresponding hydrogen bond diagram is illustrated in Figure 2a, and all detailed hydrogen bond parameters are summarized in Table 3.

The substance **2** crystallizes in the *P*2_1_/*c* space group, with half a molecule residing in each asymmetric unit. The crystal packing of **2** is primarily stabilized by strong intermolecular N1–H1...O1 (+X, 1+Y, +Z) hydrogen bonds, leading to the formation of infinite chains along the y-axis with *R*_2_^2^(10) motifs. The corresponding hydrogen bond diagram is shown in Figure 2b, and the hydrogen bond parameters are summarized in Table 3.

The substance **3** crystallizes in the *P*-1 space group, with one organic molecule and one water molecule included in each asymmetric unit. Water molecules play a pivotal role in mediating crystal packing, as they modulate the stability and ordering of the packing structure through the formation of extensive hydrogen-bonded networks. The crystal packing of **3** is dominated by strong intermolecular N1–H1...O4 and O4–H4A...O1 (−1+X, +Y, +Z) hydrogen bonds, which form infinite chains along the a-axis. Meanwhile, intermolecular O4-H4A...O3 (1−X, 1−Y, 1−Z) and O4-H4B...O2 (1−X, 2−Y, 1−Z) hydrogen bonds drive the formation of infinite chains along the b-axis. *R*_4_^4^(18), *R*_2_4(14) and *R*_4_^4^(14) hydrogen bond ring motifs are present in the network. The corresponding hydrogen bond diagram is provided in Figure 2c, and all detailed hydrogen bond parameters are summarized in Table 3.

The substance **4** crystallizes in the *I*2/*a* space group, with one organic molecule and one water molecule in each asymmetric unit. The crystal packing of **4** is governed by strong intermolecular N1–H1...O4 and O4–H4A...O1 (−1/2+X, 1−Y, +Z) hydrogen bonds, which facilitate the construction of infinite chains along the a-axis. Furthermore, intermolecular N1–H1...O4 and O4–H4B...O2 (1−X, −1/2+Y, 1/2−Z) hydrogen bonds contribute to the formation of infinite chains along the b-axis. Distinct from **3**, the hydrogen bond network of **4** contains the *R*_4_^4^(18) and *R*_8_^8^(18) hydrogen bond motifs. The aforementioned results clearly confirm that the crystal packing modes of **3** and **4** are entirely distinct. The corresponding hydrogen bond diagram is shown in Figure 2d, and all detailed hydrogen bond parameters are summarized in Table 3.

### 2.2. Electrostatic Potential and Frontier Molecular Orbitals

**Figure 3:**
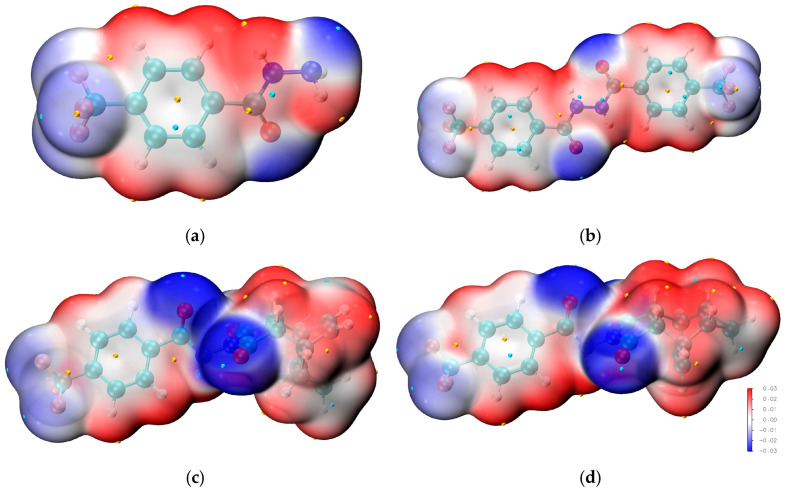
Electrostatic potential mapped on the van der Waals surfaces for **1** (**a**), **2** (**b**), **3** (**c**), and **4** (**d**). The yellow and blue spheres represent the peaks and troughs in the electrostatic potential. Four compounds share a common legend, with the units in the legend given in a.u.

Based on the single-crystal structure, geometry optimization was performed on the molecular structure, followed by the calculation of its single-point energy based on M06-2x/def2-TZVP level. Furthermore, electrostatic potential analysis was carried out utilizing the wavefunction-derived results. In Figure 3, the electrostatic potential distribution is further mapped onto the van der Waals surfaces of **1**–**4**, offering an intuitive and quantitative visualization of the intermolecular electrostatic interactions within the crystal lattice. This surface mapping technique effectively reveals the molecular electrostatic complementarity across adjacent molecules, thereby corroborating the specific roles of hydrogen bond donors and acceptors in constructing the three-dimensional supramolecular network. Specifically, the blue-colored regions on the surfaces correspond to areas with positive electrostatic potential, which are typically associated with hydrogen bond donor sites. In contrast, the red-colored domains represent regions with negative electrostatic potential, which generally act as hydrogen bond acceptor sites. The peaks and troughs in the electrostatic potential are highlighted by yellow and blue spheres, respectively. In these compounds, the oxygen atom of the amide group appears deep blue in the electrostatic potential map, indicating that it is a major nucleophilic center, while the hydrogen atom of the amide group appears deep red, indicating that it is a major electrophilic center. The fluorine atoms of the trifluoromethyl group appear lighter in color than the oxygen atoms of the amide group, indicating that the fluorine atoms are less nucleophilic and have a weaker ability to form hydrogen bonds compared to the oxygen atom.

Frontier molecular orbitals (FMOs) and their characteristic properties serve as pivotal descriptors for elucidating the mechanisms of diverse chemical reactions, as well as for pinpointing the most kinetically reactive sites within conjugated molecular systems. To gain mechanistic insights into the chemical reactivity and biological activity of the target complex, the energy levels of HOMO and LUMO, together with their energy gap, were systematically calculated. The HOMO, regarded as the outermost electron-occupied orbital, exhibits a strong propensity to donate electrons and thus acts as an electron donor; accordingly, the ionization potential of the molecule is directly correlated with the energy of the HOMO. In contrast, the LUMO functions as an electron acceptor with a high tendency to accommodate incoming electrons, and its energy level bears a direct relationship to the electron affinity of the molecule. A molecule having a lesser HOMO-LUMO gap is more polarizable and is usually related to low kinetic stability and high chemical reactivity. The HOMO-LUMO energy gaps of **1**–**4** calculated at M06-2x/def2-TZVP level are shown in Table 4. The substance **2** is a dimer of **1**. The HOMO-LUMO gaps of the two compounds are 8.1260 eV and 6.8687 eV, respectively, indicating that **2** is less stable than **1**. The substances **3** and **4** are isomers with very similar HOMO-LUMO gap values. The gap value of compound **4** is slightly larger than that of **3**, indicating that compound 4 has slightly better stability than **3**.

### 2.3. FTIR

**Figure 4:**
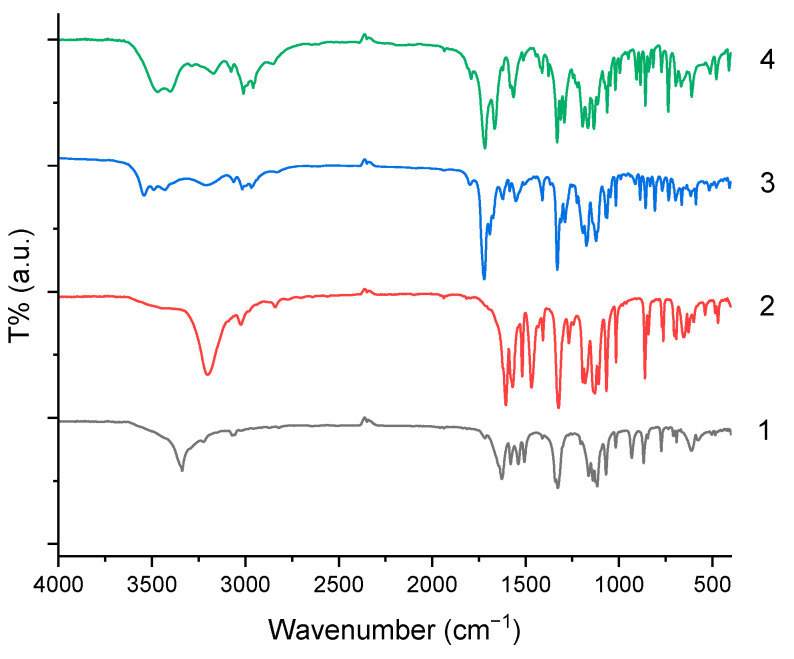
FTIR spectra with for **1**–**4** with potassium bromide pellet method.

The infrared spectra in Figure 4 of the four structurally distinct amide-containing compounds reveal characteristic patterns that align closely with their molecular architectures. The substance **1** exhibits a single hydrogen-bonded N–H stretch near 3338 cm^−1^ and a notably low carbonyl stretch at 1645 cm^−1^, consistent with a conjugated, hydrogen-bonded carbonyl in hydrazide group. The substance **2** shows only one N–H stretch (3202 cm^−1^), confirming the hydrazide-containing dicarbonyl group, and two strong carbonyl absorptions which can be assigned to the conjugated aromatic amide (1622 cm^−1^) and carbon–carbon double bond (1605 cm^−1^). In addition, intense trifluoromethyl bands between 1320 and 1100 cm^−1^ and para-substituted aromatic C–H bends near 860 and 842 cm^−1^ can be observed. The substance **3** features a rigid polycyclic imide–amide hybrid structure, displaying two N–H stretches (3431 and 3211 cm^−1^) and two carbonyl bands at 1721 cm^−1^ (aromatic amide) and 1691 cm^−1^ (hydrogen-bonded lactam carbonyl in the strained tetracyclic core), along with olefinic =C–H stretch (3016 cm^−1^) from the endocyclic double bond. The substance **4** contains both an aromatic amide and a saturated bicyclic lactam, giving rise to two resolved N–H stretches (3471 and 3402 cm^−1^) and two carbonyl bands at 1717 cm^−1^ (benzamide) and 1666 cm^−1^ (strongly H-bonded imidazolidinone lactam); its spectrum also shows clear signatures of the para-CF_3_-phenyl group and aliphatic C–H deformations from the thieno-fused saturated ring system. Across all four compounds, the positions and multiplicities of ν(N–H), ν(C=O), ν(CF_3_), and γ(C–H) bands provide definitive evidence for their respective functional groups and substitution patterns.

### 2.4. Hirshfeld Surface Analysis

Hirshfeld surface analysis was performed to explore the intermolecular contacts in the crystal structures. As a robust graphical tool, this analysis enables the investigation and quantification of diverse intermolecular interactions within crystal lattices. The quantification of the intermolecular interactions was achieved by generating the Hirshfeld surfaces and the two-dimensional fingerprint plots [27] using CrystalExplorer [28] software (version 25.09). In Hirshfeld surface analysis, the disorder parts of the crystal structures with low site occupancy factors (SOF) were removed.

The Hirshfeld surface of these four compounds were mapped with *d*_norm_ (from −0.5 to 1.0) in CrystalExplorer. The larger red spots visualized on the *d*_norm_ maps indicate the presence of strong hydrogen bond contacts, and the smaller red spots correspond to the existence of weak hydrogen bond contacts. The remaining white regions imply the existence of certain other relatively weak interactions, while the blue regions indicate a near-complete lack of intermolecular interactions. Two-dimensional fingerprint plots are presented for each compound and Table 5 illustrates the relative contributions of various intermolecular contacts to the Hirshfeld surface area of each compound.

As presented in Table 5, six types of intermolecular contacts can be identified in the crystal structure, with each corresponding to a distinct weak interaction. Specifically, the N...H/H...N contacts are attributed to hydrogen bonds with nitrogen as the acceptor; the O...H/H...O contacts arise from hydrogen bonds with oxygen as the acceptor; the C...H/H...C contacts correspond to C-H...π interactions formed between alkyl/aryl C-H groups and aromatic π-systems; the C...C contacts are characteristic of π-π interactions between adjacent aromatic rings; the F...H/H...F contacts refer to hydrogen bonds with fluorine as the acceptor; and the F...F contacts are assigned to F...F interactions.

Across the four target compounds, the percentage of N…H/H…N intermolecular contacts is consistently low (from 4.3% to 0.2%). This observation suggests that there are merely a few nitrogen-acceptor hydrogen bonds present in their respective crystal architectures, and these hydrogen bonds exhibit weak interaction strength due to the low proportion of relevant contacts. Similarly, the π-π interactions are also weak, which can be deduced from the C...C percentage (from 1.1% to 1.3%). Although benzene rings are present in all four crystal structures, effective π-π stacking is not observed.

**Figure 5:**
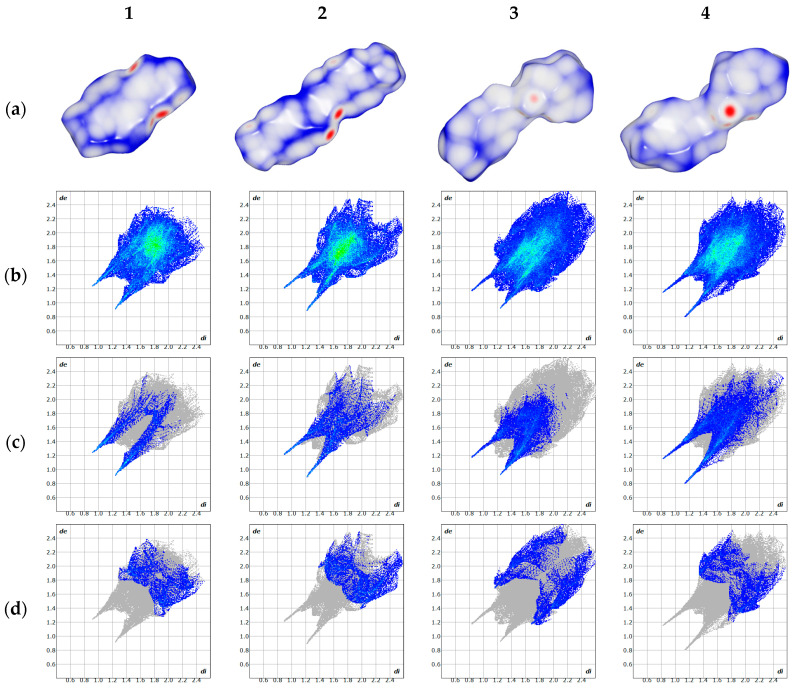

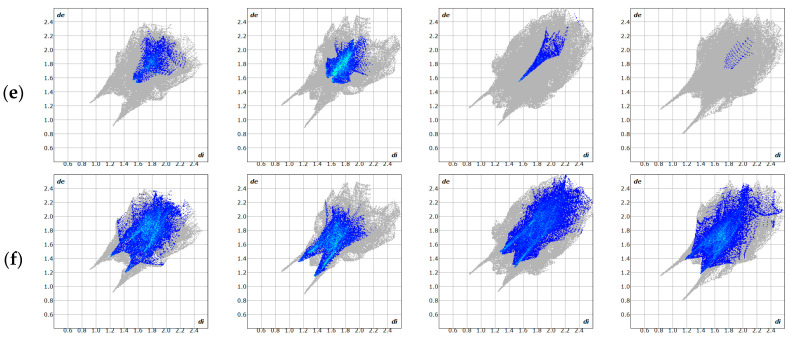
Hirshfeld surfaces of **1**, **2**, **3**, and **4** mapped with dnorm (**a**). Full 2D-fingerprint plots (**b**) and important contacts plots [O...H (**c**), C...H/H...C (**d**) and F...F (**e**) F...H/H...F (**f**)] for **1**–**4** where *d*_e_ and *d*_i_ represent the *y*- and *x*-axis parameters.

Unlike the aforementioned two types of contacts, the O…H/H…O contacts (from 11.6% to 21.2%) account for a remarkably high proportion among all intermolecular interactions. This indicates the presence of prominent oxygen-acceptor hydrogen bonds in the crystal structures, which correspond to the hydrogen bond network described in Section 2.1. In Figure 5c, each O...H/H...O map shows two spikes. The spikes are indicative of strong hydrogen bonding interactions. The bond lengths of these hydrogen bonds are equivalent to the sum of *d*_e_ and *d*_i_, specifically around 2.1 Å. The C…H/H…C contacts are highly similar to the F…H/H…F contacts. As shown in Table 5, both types of contacts account for a considerably high proportion; however, the sum of *d*_e_ and *d*_i_ corresponding to the spike values is extremely low, as indicated in Figure 5d,f. This demonstrates that although the C-H…π interactions and F-acceptor hydrogen bonds are present in high proportions, their interaction strengths are relatively weak.

Unlike the aforementioned intermolecular contacts, the proportion of F…F contacts varies remarkably across the four compounds: it is relatively high in **2** (16.3%), low in 1 (6.7%) and **3** (2.3%), and nearly zero in **4**. This indicates a high proportion of F…F interactions in **2**, low proportions in **1** and **3**, and almost the absence of F…F interactions in **4.** In Figure 5e, the sum of *d*_e_ and *d*_i_ corresponding to the spike values are about 3.2 Å. The F…F interaction distance of 3.2 Å in our system is longer than the sum of van der Waals radii of F atoms, indicating that the F…F interactions in the above-mentioned compounds are too weak to neglect.

Taken together, the interaction proportions of the compounds listed in the above table are quite close, indicating that their intermolecular interaction modes are highly analogous. To quantitatively evaluate the strength of these intermolecular interactions, a packing energy analysis will be presented in subsequent Section 2.7.

### 2.5. Phase Analysis

**Figure 6:**
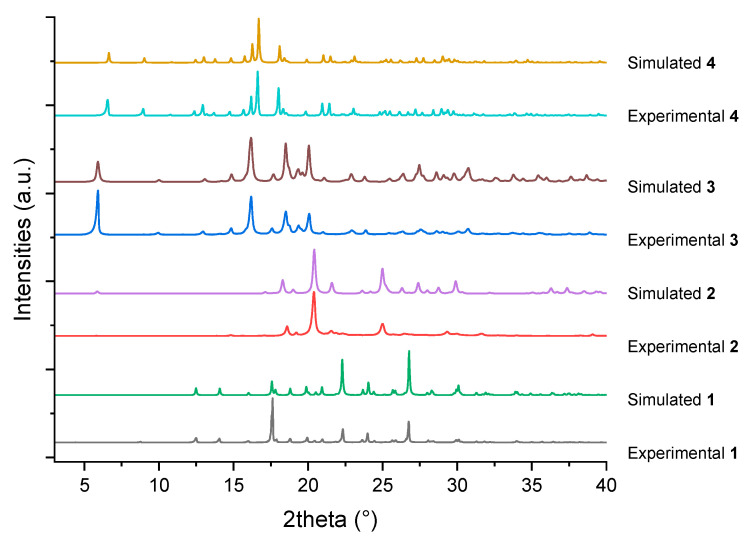
Experimental and simulated PXRD patterns at a scan speed of 5°/min.

The PXRD data were collected and analyzed to assess the crystallinity and identify the phase of the sample. The as-obtained crystals were retrieved from the solution, air-dried under ambient conditions, and subsequently ground into a fine homogeneous powder using a pre-cleaned agate mortar and pestle prior to characterization. The measured powder diffraction pattern was compared with the one simulated from the single-crystal structure, and the results are shown in Figure 6 below. The simulated powder diffraction pattern was calculated using the Mercury [29] (Build 445489) software, with the full width at half maximum (FWHM) set to 0.1° during the calculation. The experimental PXRD patterns of each compound exhibited good agreement with the simulated one, indicating that the samples were highly crystalline and free of detectable impurities or amorphous phases.

### 2.6. Melting Points

**Figure 7:**
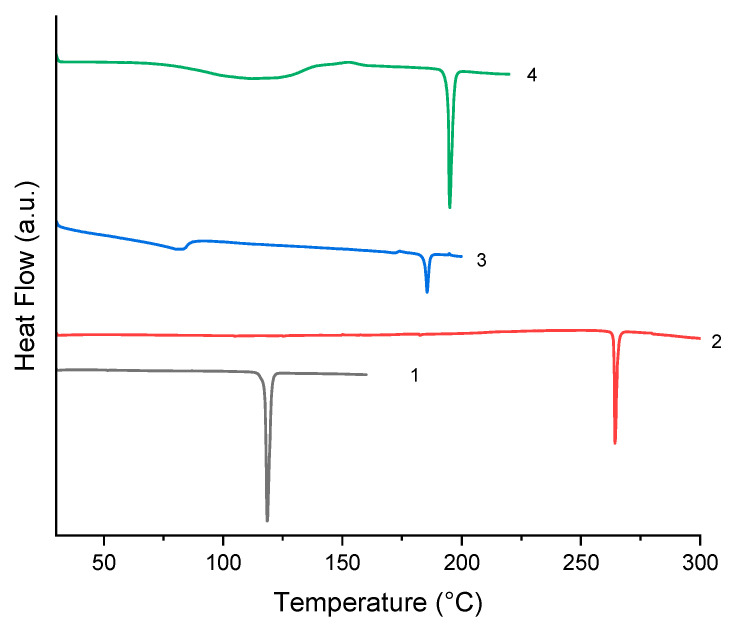
DSC profiles for **1**–**4** with a heating rate of 10 °C/min.

In this study, DSC was employed to investigate the thermal properties and stability of these compounds. The DSC profiles of these compounds are shown in Figure 6. The four compounds all display a single sharp endothermic peak, which arises from the melting of the compounds. The onset values of the endothermic peaks are shown in Table 6. In addition to the onset temperature, Table 6 also provides information on density, packing index and packing energy calculated in Section 2.7. In addition to the aforementioned sharp endothermic peak, **3** and **4** each exhibit a broad endothermic peak arising from the loss of water with peak values of 83.17 °C and 111.83 °C, respectively. The substances **3** and **4** possess similar structures, and thus their melting points are extremely close. Only a difference of approximately 10 °C is observed in Figure 7. The melting point of **3** is slightly lower than that of **4**, indicating that **4** exhibits higher stability than **3**. **2** is the dimer of compound **1**, and the two exhibit a significant difference in melting points. This difference can be accounted for by the lengths of strong hydrogen bonds: those of compound **1** are all above 3 Å, while those of compound **2** are below 3 Å. Generally speaking, the shorter the hydrogen bond, the higher its bond energy. In addition to the hydrogen bond lengths, the origin of the melting point difference is further confirmed by lattice energy calculations and analysis in the subsequent Section 2.7.

### 2.7. Packing Energies

As mentioned in Section 2.2, the proportions of various intermolecular interactions among these four compounds are quite close. To quantitatively assess the strength of these intermolecular forces, calculations of the intermolecular interactions have been performed. The pairwise interaction energies were computed using the CE-1p [30] energy model in CrystalExplorer. Compared to old CE-B3LYP model [31], the new model incorporates an improved treatment of dispersion interactions and polarizabilities using the exchange-hole dipole model (XDM), along with the use of effective core potentials (ECPs), facilitating application to molecules containing elements across the periodic table (from H to Rn) [30]. The total interaction energy of the CE-1p energy model is calculated as a sum of physically motivated components:

*E*_tot_ = *E*_coul_ + *k*_rep_ *E*_rep_ + *E*_exch_ + *k*_pol_ *E*_pol_ + *E*_disp_ (1)

where *E*_coul_ represents Coulombic (electrostatic) interactions, *E*_rep_ is repulsion from orbital orthogonalization, *E*_exch_ accounts for exchange interactions, *E*_pol_ is the polarization energy, and *E*_disp_ captures dispersion interactions. The CE-1p model uses a single empirical parameter *k* = 0.78 that scales both the repulsion and polarization terms (*k*_rep_ = *k*_pol_ = 0.78).

It is clarified that the melting process actually occurs for the dehydrated solids, rather than the hydrated crystalline forms characterized by X-ray diffraction. Therefore, the comparison of melting points between **3** and **4** is indeed based on the lattice energies of the dehydrated phases. The total results of the interaction energy calculations are summarized in Supplementary Information. The final results of the packing energies are summarized in Table 6. In addition to packing energies, the crystal packing was quantitatively evaluated using the “Calc K.P.I.” function in the PLATON [32,33] software (version 240625). As can be seen from the table, the packing energies of **3** and **4** are extremely close to each other, with the absolute value of the packing energy of **3** being smaller than that of **4**. Notably, the differences in packing energies are in good correspondence with the variations in melting points. In contrast, there is a significant disparity in the packing energies between **1** and **2**, with a difference of approximately 50 kJ/mol. Such a substantial energy difference gives rise to the remarkable variation in their melting points. A comparative analysis of the four compounds reveals that their melting points follow the descending order of **2** > **4** > **3** > **1**. Notably, this sequence exhibits a strict one-to-one correspondence with the order of the absolute values of their packing energies. In addition, the data demonstrate that **2** possesses the highest density and packing ratio, accompanied by the highest melting point. In contrast, although **1** ranks second in terms of density and packing ratio, it shows the lowest melting point. These results indicate that there is no inevitable correlation between the density, packing ratio of crystals and their melting points; thus, the former two cannot be regarded as decisive factors for determining melting point variations. On the contrary, the magnitude of packing energy can serve as a reliable criterion for explaining the differences in the melting points of the compounds [34].

### 2.8. In Silico Toxicity Assessment

In silico toxicological assessment requires the employment of at least two complementary computational approaches, as this strategy enables reliable prediction of bacterial mutagenicity assay outcomes. Specifically, the bacterial mutagenicity in silico of **1**–**3** was evaluated in compliance with International Council for Harmonisation of Technical Requirements for Pharmaceuticals for Human Use (ICH) M7 guidelines and U.S. Food and Drug Administration’s (FDA) guidelines [35] via QSAR models [36] integrated within Derek Nexus [37] software (version 6.4.2 with Derek knowledge bases version 2024 1.0) and Sarah Nexus [38] software (version 5.0.0 with Sarah model version 1.12). The substances **1** and **3** were predicted to be plausible, indicating that their molecular structures contain substructures matching the built-in alert rules for potential toxicity, thus suggesting a reasonable level of genotoxic risk. In contrast, **2** was classified as inactive in Derek, meaning no toxic alerting structures were identified and no obvious toxicity risk was predicted. Consistently, all three compounds (**1**–**3**) yielded negative predictions in Sarah. These results statistically confirm that the compounds are non-mutagenic at the predicted endpoint, with high confidence supported by fragment-based structural analysis and large-scale comparative toxicological data.

For the comprehensive assessment under ICH M7 guidelines: **2** showed consistent predictions (inactive in Derek Nexus and negative in Sarah Nexus), demonstrating a very low genotoxic risk that allows for streamlined regulatory evaluation without additional in vitro assays such as the Ames test. Consequently, **1** and **3** were formally assigned to ICH M7 Class 3, a category encompassing impurities with structure-unrelated mutagenic structural alerts and a lack of reliable mutagenicity data for conclusive assessment. This classification was determined following comprehensive in silico toxicology evaluations and expert review, as the predictive results failed to provide sufficient evidence to exclude their potential genotoxic risk. On this basis, additional testing is recommended for **1** and **3**.

## 3. Materials and Methods

### 3.1. Crystallization

**1**–**4** used in this study were purchased from Macklin Inc. (Rochelle, IL, USA). All reagents were used directly without further purification. Approximately 10 mg of each compound was accurately weighed, and 3 mL of analytical grade ethanol (Sinopharm Chemical Reagent Co., Ltd., Shanghai, China) was added, followed by ultrasonication to achieve complete dissolution. The resulting solutions were placed in a fume hood for standing, and the target crystals were obtained after one week. Among them, **4** crystallized as block-shaped crystals, **1** and **3** as plate-shaped crystals, and **2** as needle-shaped crystals.

### 3.2. Single-Crystal X-Ray Diffraction

Air-stable single crystals were selected to be mounted on thin glass fibers. Data were collected using an XtaLAB Synergy R, DW system, HyPix diffractometer (Rigaku Corporation, Tokyo, Japan) operating at room temperature with CuKα (λ = 1.54178 Å) radiation. CrysAlis^Pro^ [39] software package version 1.171.42.92a was used for data reduction, scaling, and absorption. The programs integrated into Olex2 [40] were used for structure solution, refinement, and analysis. The crystal structures were solved using the ShelXT [41] program employing intrinsic phasing methods, and refined by full-matrix least-squares techniques with ShelXL [42] version 2019.

All non-hydrogen atoms were refined anisotropically. The trifluoromethyl group at the end of the molecule underwent disorder refinement with the PART instruction [43]. The occupancies were refined freely. Some instructions (SIMU and DFIX) were performed to obtain a better model for the trifluoromethyl groups.

The positions of hydrogen atoms on carbon atoms were calculated geometrically and refined using the riding model. Their coordinates and displacement parameters were constrained to ride on the carrier atom with C–H = 0.98 Å and *U*_iso_(H) = 1.2*U*_eq_(C) for alkyl H atoms, and C–H = 0.93 Å and *U*_iso_(H) = 1.2*U*_eq_(C) for aromatic H atoms.

Active hydrogen atoms on oxygen atoms and nitrogen atoms were located from difference Fourier maps inspection and freely refined with *U*_iso_(H) = 1.5*U*_eq_(O) or *U*_iso_(H) = 1.5*U*_eq_(N). The distances between the active hydrogen atoms and non-carbon atoms were restrained to 0.85 Å for hydrazide group and water molecules.

CCDC No. 2494747, 2494748, 2494749, and 2502862 contain the supplementary crystallographic data for **1**–**4**, respectively. These crystallographic information files (CIF) can be obtained free of charge via www.ccdc.cam.ac.uk (accessed on 26 November 2025) or by e-mailing data_request@ccdc.cam.ac.uk, or by contacting The Cambridge Crystallographic Data Centre, 12 Union Road, Cambridge, CB2 1EZ, UK; fax: +44-1223-336033.

### 3.3. PXRD

PXRD analyses were performed on a Rigaku SmartLab diffractometer (Rigaku Corporation, Tokyo, Japan) using CuKα radiation. The samples were measured at ambient temperature in a 2θ range of 3–40°, at a scan speed of 5 °/min. PXRD data were investigated using Jade software package (version 6.0).

### 3.4. DSC

DSC was conducted using a Mettler Toledo DSC 3 instrument (Greifensee, Switzerland) with aluminum crucibles equipped with perforated lids. The measurements were carried out under an inert nitrogen atmosphere at a flow rate of 20 mL/min, with a heating rate of 10 °C/min. An indium metal standard was used for temperature reference.

### 3.5. Fourier Transform Infrared Spectroscopy (FT-IR)

FT-IR spectra were acquired on a PerkinElmer Frontier FT-IR spectrometer (PerkinElmer Inc., Waltham, MA, USA). For the pretreatment of FT-IR samples, the standard potassium bromide pellet method was employed. Data were recorded with a resolution of 4 cm^−1^, on average, of 16 scans between 4000 cm^−1^ and 400 cm^−1^.

### 3.6. Hirshfeld Surfaces and Packing Enegies

These calculations were performed with CrystalExplorer (version 25.09) software. The Hirshfeld surfaces were mapped as reported [44]. The Hirshfeld surfaces, intermolecular interactions [45], and their reciprocal fingerprints [46] were determined as reported [47,48].

The interaction energies were calculated based on the CrystalExplorer’s built-in CE-1p model. The packing energies could be obtained by summing the energies of all intermolecular interactions using the method reported in the literature [49]. During calculations of **3** and **4**, the water molecules in the lattice were removed to obtain the dehydrated phases. Subsequently, the interaction energy of this anhydrous phase was calculated, from which the packing energy was then derived.

All the diagrams were directly generated and exported via CrystalExplorer software.

### 3.7. Theoretical Study

All theoretical calculations were performed with ORCA 6.1.1 [50,51,52], and the corresponding input files were generated using the CrystalExplorer software. Initially, geometry optimization was carried out at the B3LYP/def2-SVP level of theory. Subsequent single-point energy calculations were conducted at the M06-2x/def2-TZVP level to generate wave functions for subsequent analysis. ESP surfaces and molecular orbital were generated with Multiwfn (version 3.8) [53,54,55,56] and visualized in VMD [57] to assist in the interpretation of sensitivity-related behavior as reported [16,58].

## 4. Conclusions

This study systematically characterized tecovirimat (**4**) and three representative process-related substances (**1**–**3**) using a multi-technique approach, including single-crystal XRD, PXRD, DSC, Hirshfeld analysis, packing energy calculations, and in silico toxicity assessment. The single-crystal structures of **1**–**3** were elucidated for the first time, revealing distinct space groups driven by diverse hydrogen bonding networks. The study confirmed that the melting point order (**2** > **4** > **3** > **1**) correlates strictly with the magnitude of packing energies, establishing packing energy as a reliable predictor of thermal stability in this system. Furthermore, toxicological assessment classified **2** as having low genotoxic potential, whereas **1** and **3** (ICH M7 Class 3) require further in vitro investigation. These findings provide a comprehensive structural and safety framework for the quality control and process optimization of tecovirimat. This research enriches tecovirimat-related crystallographic data, establishes an analytical framework for antiviral drug impurities, and provides guidance for optimizing tecovirimat synthesis, improving purity, and ensuring safety. Future work may focus on in vitro genotoxicity testing of these compounds.

## Figures and Tables

**Table 1:** Crystal data and details of structure refinement.

Parameters	1	2	3	4
CCDC No.	2494747	2494748	2494749	2502862
Empirical formula	C_8_H_7_F_3_N_2_O	C_16_H_10_F_6_N_2_O_2_	C_19_H_15_F_3_N_2_O_3_·H_2_O	C_19_H_15_F_3_N_2_O_3_·H_2_O
Formula weight	204.16	376.26	394.34	394.34
Temperature/K	297	297	297	303
Crystal system	orthorhombic	monoclinic	triclinic	monoclinic
Space group	*Pbca*	*P*2_1_/*c*	*P*-1	*I*2/*a*
*a*/Å	9.8807(3)	5.1461(2)	6.57437(15)	12.8946(2)
*b*/Å	8.8602(3)	4.8934(2)	9.5136(3)	10.53859(19)
*c*/Å	20.0898(6)	30.1328(14)	15.6987(5)	26.6710(4)
*α*/°	90	90	105.655(3)	90
*β*/°	90	90.026(4)	96.308(2)	94.3614(13)
*γ*/°	90	90	103.324(2)	90
Volume/Å^3^	1758.77(9)	758.81(6)	904.37(4)	3613.85(10)
Z	8	2	2	8
*ρ*_calc_/g cm^−3^	1.542	1.647	1.448	1.450
*μ*/mm^−1^	1.304	1.420	1.056	1.057
*F*(000)	832.0	380.0	408.0	1632.0
Crystal size/mm	0.4 × 0.4 × 0.05	0.5 × 0.1 × 0.1	0.4 × 0.4 × 0.08	0.6 × 0.3 × 0.3
Radiation	Cu *Kα*	Cu *Kα*	Cu *Kα*	Cu *Kα*
2*Θ* range for data collection/°	8.804 to 152.81	5.866 to 153.23	5.948 to 152.812	6.648 to 153.174
Index ranges	−12 ≤ h ≤ 12 −10 ≤ k ≤ 7 −23 ≤ l ≤ 25	−6 ≤ h ≤ 6 −5 ≤ k ≤ 4 −37 ≤ l ≤ 37	−8 ≤ h ≤ 7 −11 ≤ k ≤ 11 −19 ≤ l ≤ 19	−16 ≤ h ≤ 16 −9 ≤ k ≤ 12 −29 ≤ l ≤ 33
Reflections collected	8182	7446	17,243	16,571
Independent reflections	1767	1511	3665	3616
*R*_int_	0.0610	0.0524	0.0386	0.0236
*R*_sigma_	0.0401	0.0382	0.0263	0.0170
Data/restraints/parameters	1767/3/165	1511/1/121	3665/10/290	3616/2/264
Goodness-of-fit on *F*^2^	1.058	1.075	1.056	1.093
*R*_1_, *w*R_2_ [*I* ≥ 2σ (*I*)]	*R*_1_ = 0.0411 *w*R_2_ = 0.1186	*R*_1_ = 0.0746 *w*R_2_ = 0.2088	*R*_1_ = 0.0449 *w*R_2_ = 0.1282	*R*_1_ = 0.0490 *w*R_2_ = 0.1372
*R*_1_, *w*R_2_ [all data]	*R*_1_ = 0.0484 *w*R_2_= 0.1276	R1 = 0.0860 *w*R_2_ = 0.2165	*R*_1_ = 0.0525 *w*R_2_ = 0.1362	*R*_1_ = 0.0530 *w*R_2_ = 0.1413
Largest diff. peak/hole/e Å^−3^	0.16/−0.16	0.37/−0.25	0.19/−0.18	0.64/−0.53

**Table 2:** Comparison of key ring dimensions between the four compounds (Å, °).

Bond Length	1	2	3	4
O1–C8	1.2331(17)	1.233(4)	1.2155(17)	1.220(2)
N1–N2/N1^1−X,2−Y,−Z^	1.407(2)	1.394(5)	1.3809(16)	1.3853(18)
N1–C8	1.3329(19)	1.336(4)	1.3574(19)	1.342(2)
O2–C9		data	1.2070(18)	1.210(2)
O3–C12		data 1	1.2062(17)	1.195(2)
N2–C9			1.383(2)	1.381(2)
N2–C12			1.3952(17)	1.398(2)
Angle				
C3–C4–C8–N1	13.0(2)	32.8(5)	10.4(2)	9.8(3)

**Table 3:** Hydrogen bond parameter for four compounds (Å, °).

Compounds	D	H	A	d(D-H)	d(H-A)	d(D-A)	D-H-A
**1**	N1	H1	O1 ^i^	0.860(16)	2.173(17)	3.0058(16)	163(2)
	N2	H2A	O1 ^ii^	0.875(16)	2.323(17)	3.180(2)	166(2)
**2**	N1	H1	O1 ^iii^	0.85(2)	2.12(2)	2.942(4)	162(4)
**3**	N1	H1	O4	0.868(15)	2.007(16)	2.8584(17)	166(2)
	O4	H4A	O1 ^iv^	0.849(17)	2.33(2)	2.9710(17)	132(2)
	O4	H4A	O3 ^v^	0.849(17)	2.37(2)	2.9672(17)	128(2)
	O4	H4B	O2 ^vi^	0.847(17)	2.166(17)	3.0058(18)	171(2)
**4**	N1	H1	O4	0.86(2)	1.94(2)	2.767(2)	163(2)
	O4	H4A	O1 ^vii^	0.851(17)	1.937(18)	2.7853(19)	174(3)
	O4	H4B	O2 ^viii^	0.859(17)	2.042(19)	2.886(2)	167(3)

Symmetry codes: i: 1/2+X,1/2−Y,1−-Z; ii: 3/2−X,1/2+Y,+Z; iii: +X,1+Y,+Z; iv: −1+X,+Y,+Z; v: 1−X,1−Y,1−Z; vi: 1−X,2−Y,1−Z; vii: −1/2+X, 1−Y, +Z; viii: 1−X, −1/2+Y, 1/2−Z.

**Table 4:** HOMO-LUMO energy gaps of **1**–**4**.

Interactions	1	2	3	4
*E*_HOMO_ (eV)	−9.2117	−8.4159	−8.9269	−8.9876
*E*_LUMO_ (eV)	−1.0857	−1.5472	−1.1825	−1.1839
Energy Gap (eV)	8.1260	6.8687	7.7444	7.8037

**Table 5:** Hirshfeld surface area of each compound (%).

Interactions	1	2	3	4
H...H	13.5	7.3	25.3	26.5
N...H/H...N	4.3	2.8	0.5	0.2
O...H/H...O	11.6	15.3	16.5	21.2
C...H/H...C	10.3	14.2	11.1	8.2
C...C	1.1	1.4	0.2	1.3
F...H/H...F	27.9	20.7	25.8	23.0
F...F	6.7	16.3	2.3	0.1

**Table 6:** Calculated density from CIF, onset value from DSC, packing index from PLATON and packing energy from CrystalExplorer.

Compounds	Density	Onset Value	Packing Index	Packing Energy
**1**	1.542 g/cm^3^	117.48 °C	67.8%	−91.4 kJ/mol
**2**	1.647 g/cm^3^	263.72 °C	70.6%	−142.5 kJ/mol
**3**	1.448 g/cm^3^	184.34 °C	67.1%	−116.5 kJ/mol
**4**	1.450 g/cm^3^	194.10 °C	67.2%	−119.8 kJ/mol

## Data Availability

Dataset available on request from the authors.

## Supplementary Materials

The following supporting information can be downloaded at: https://www.mdpi.com/article/10.3390/molecules31030502/s1, Figures S1–S12: Packing diagram of **1**–**4**. Figures S13–S16: Hirshfeld surfaces analysis results for **1**–**4**; Figures S17–S20: HOMO and LUMO of **1**–**4**. Figures S21–S24: Original FTIR spectra of **1**–**4**. Figure S25–S28: Original DSC profiles of **1**–**4**. Tables S1–S30: Additional X-ray crystallography data; Tables S31–S34: Detailed interaction energy calculation results.
