# Supplementary material for: Tecovirimat-Related Substance: Characterization, Hirshfeld Analysis, Theoretical Study, In Silico Toxicity Assessment
Source: Molecules. 2026 Jan 31;31(3):502. doi: 10.3390/molecules31030502 (PMC12899545; doi:10.3390/molecules31030502)
Supplement: Supplementary file 1 [file molecules-31-00502-s001.zip › molecules-4071429-supplementary.pdf]

Supporting Information

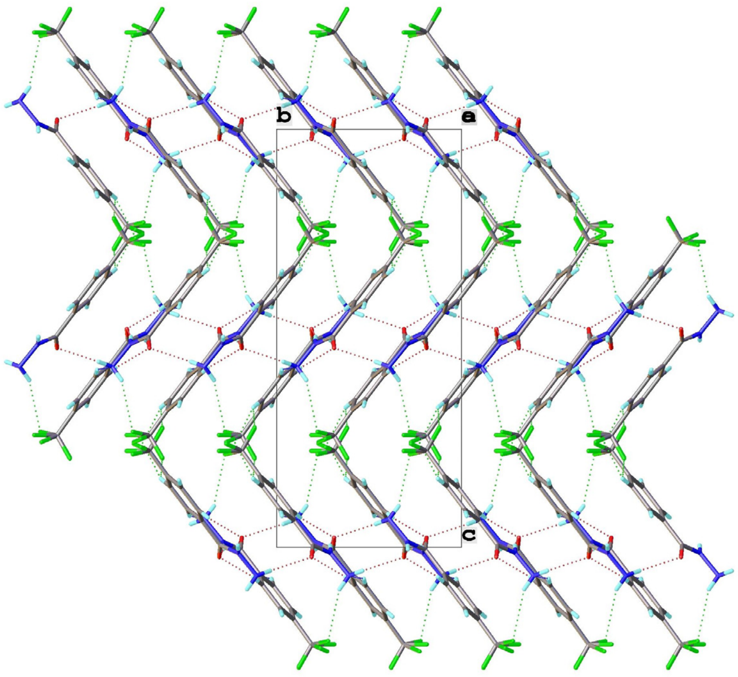

Figure S1. Packing diagram along *a* axis of **1**

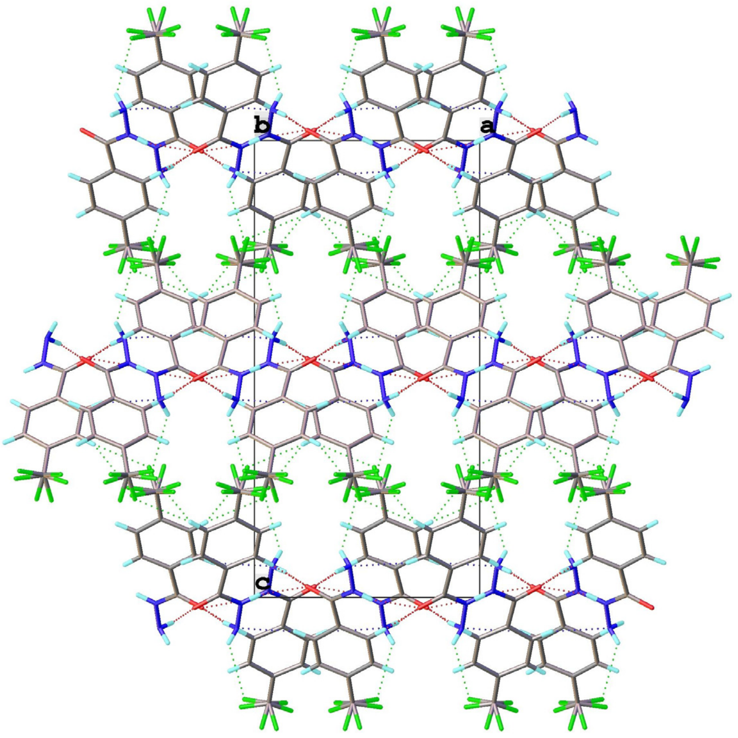

Figure S2. Packing diagram along *b* axis of **1**

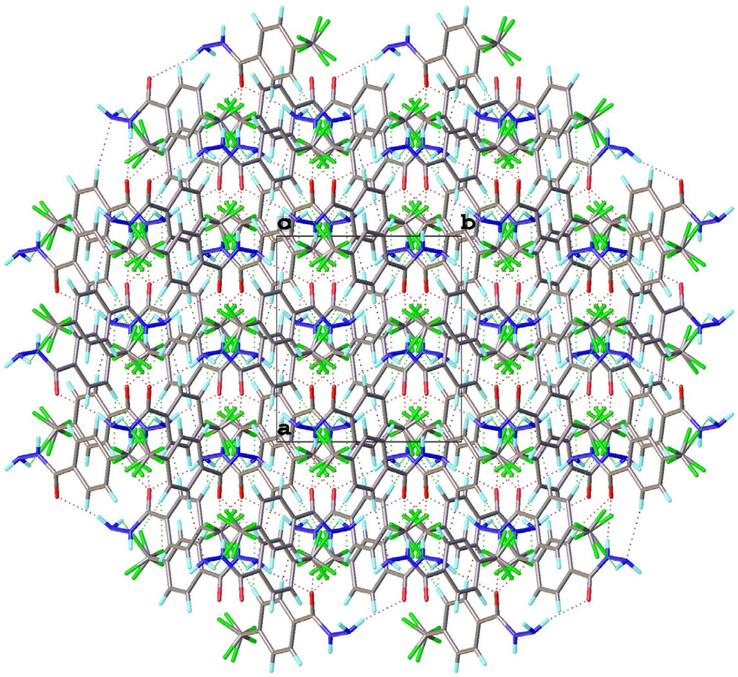

Figure S3. Packing diagram along *c* axis of **1**

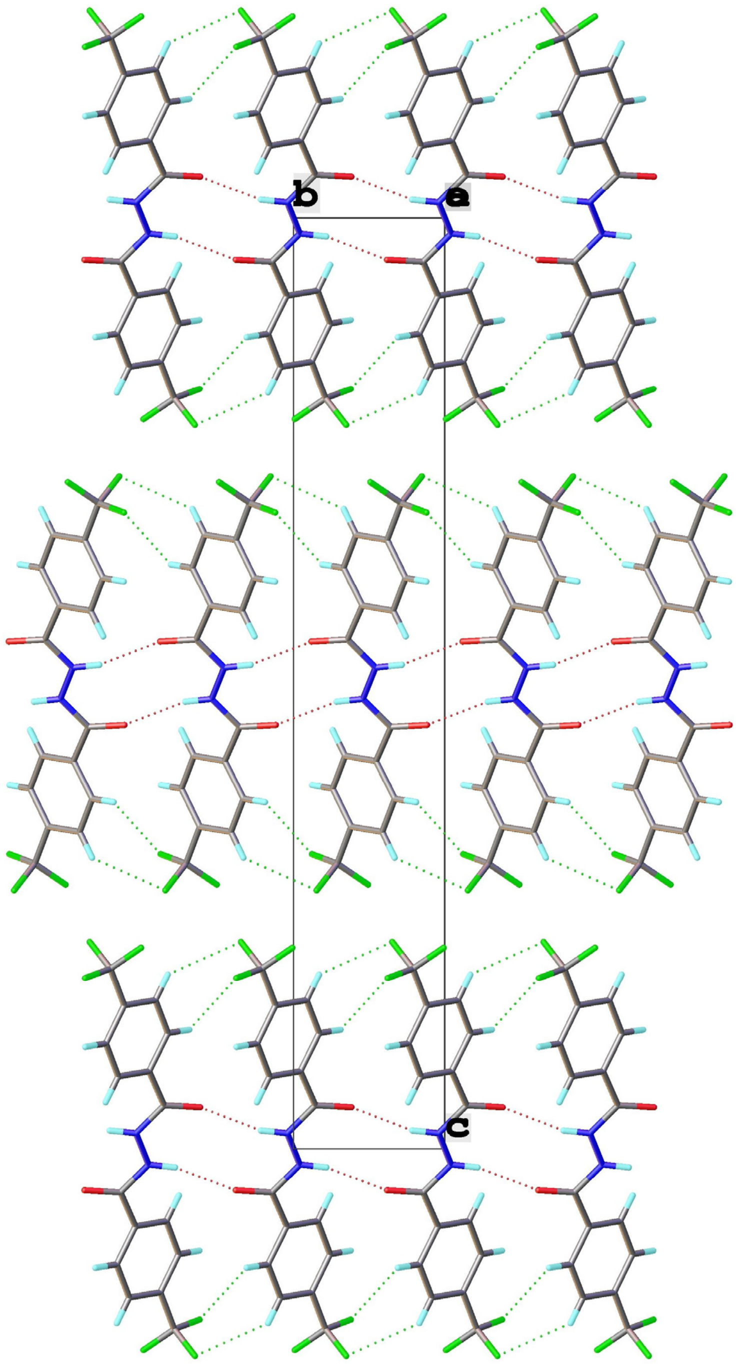

Figure S4. Packing diagram along *a* axis of **2**

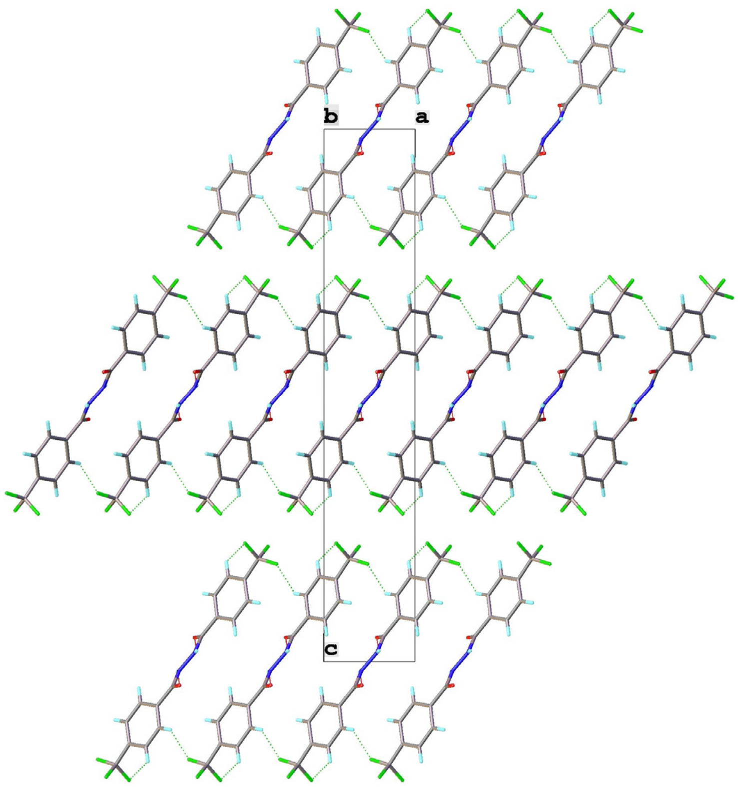

Figure S5. Packing diagram along *b* axis of **2**

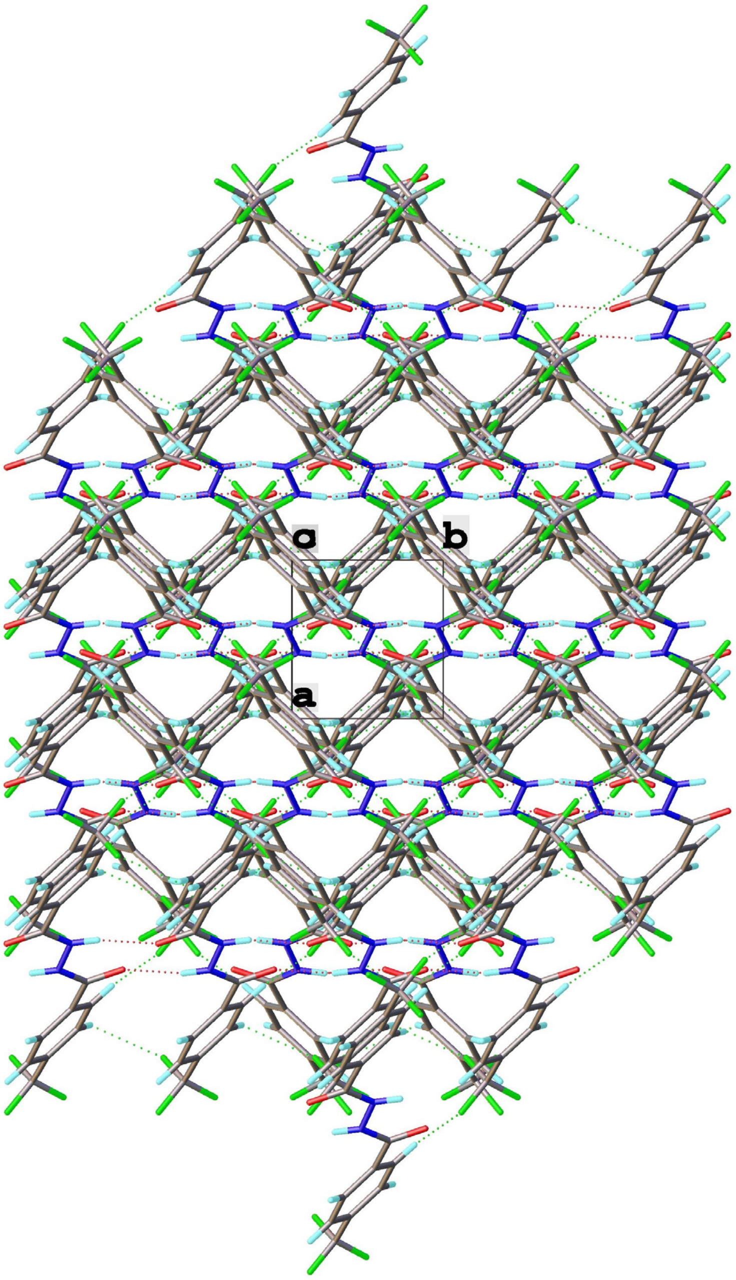

Figure S6. Packing diagram along *c* axis of **2**

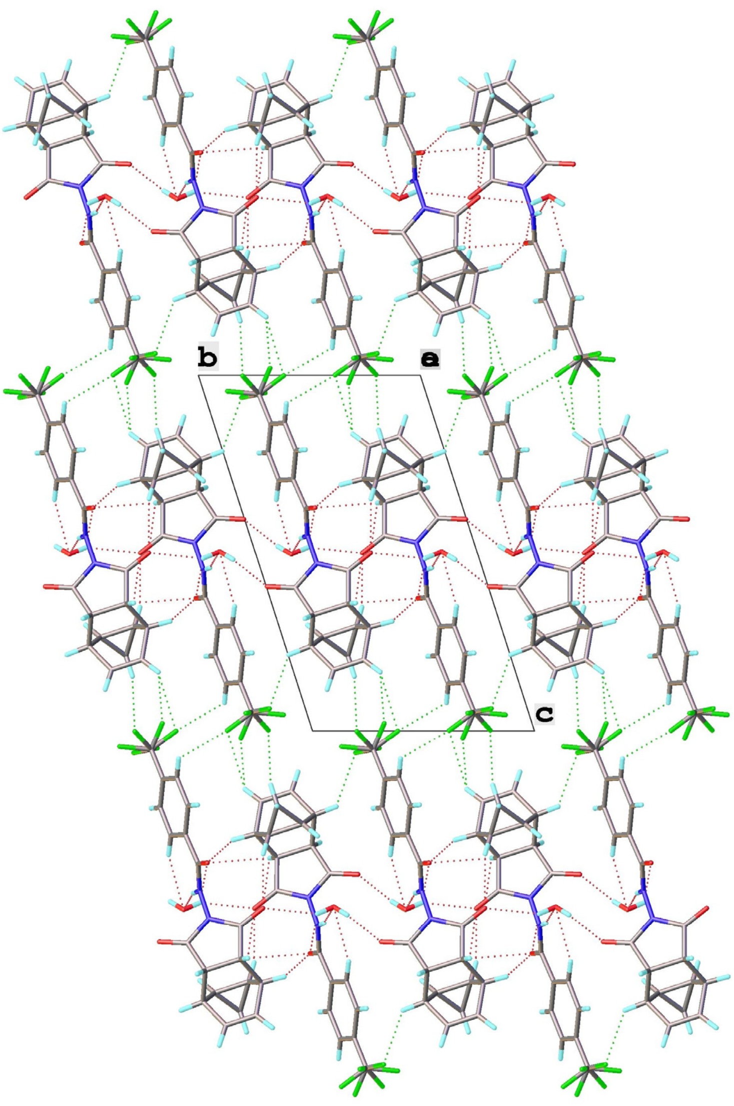

Figure S7. Packing diagram along *a* axis of **3**

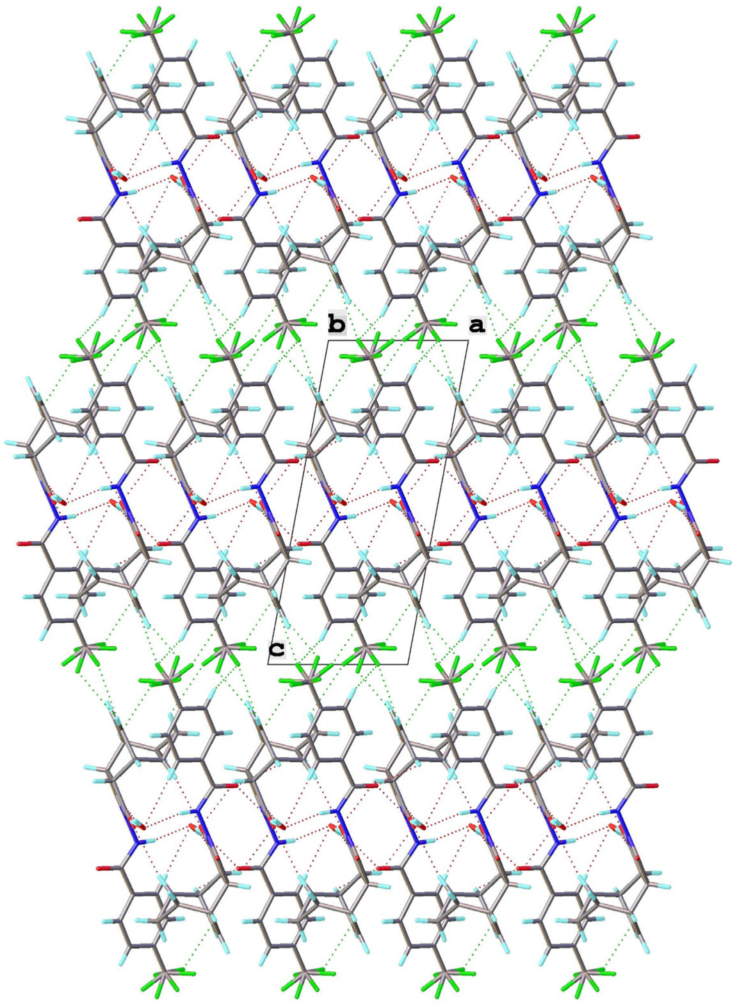

Figure S8. Packing diagram along *b* axis of **3**

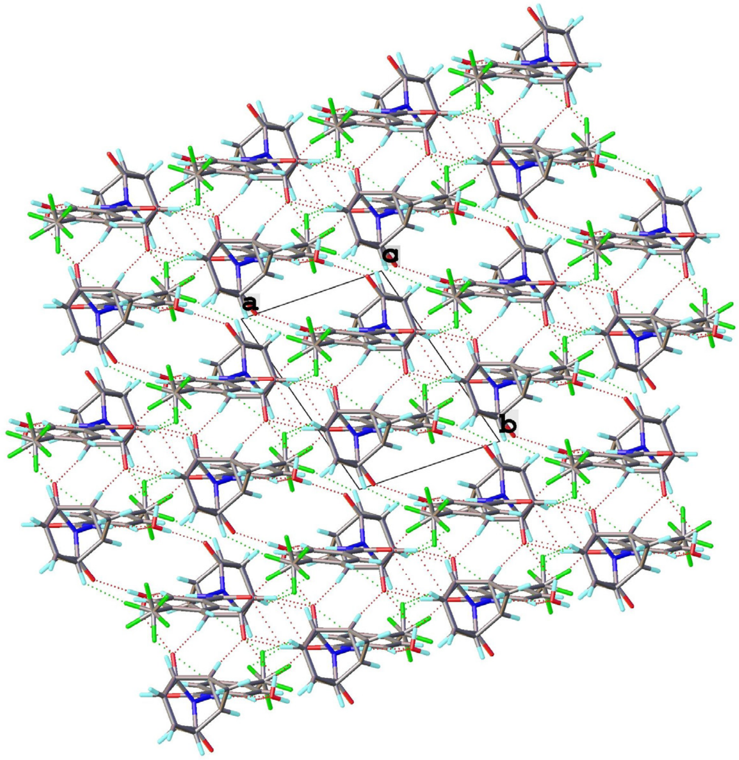

Figure S9. Packing diagram along *c* axis of **3**

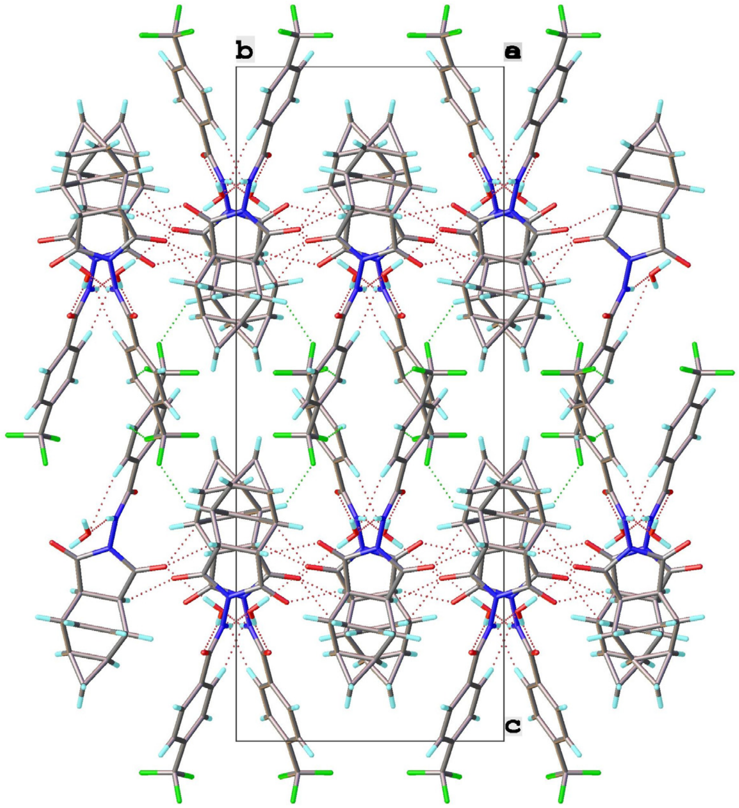

Figure S10. Packing diagram along *a* axis of **4**

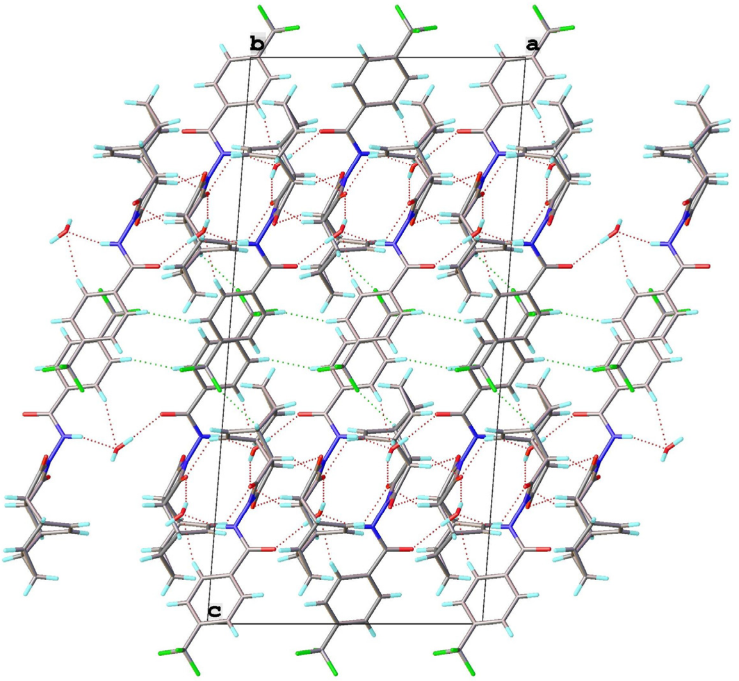

Figure S11. Packing diagram along *b* axis of **4**

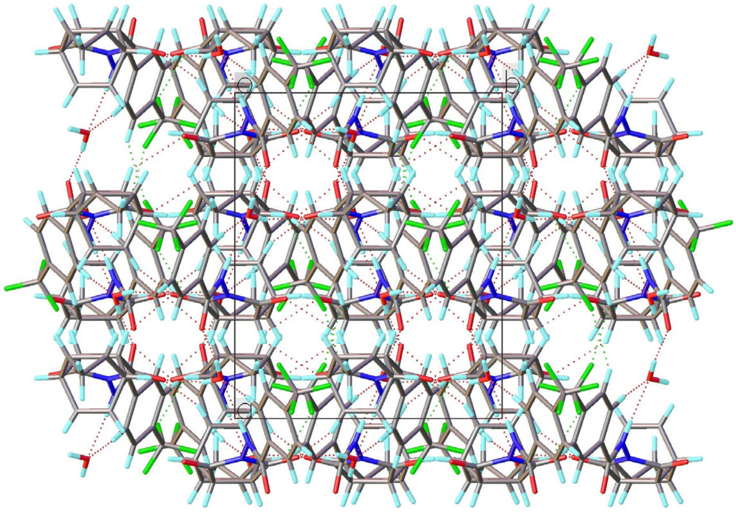

Figure S12. Packing diagram along *c* axis of **4**

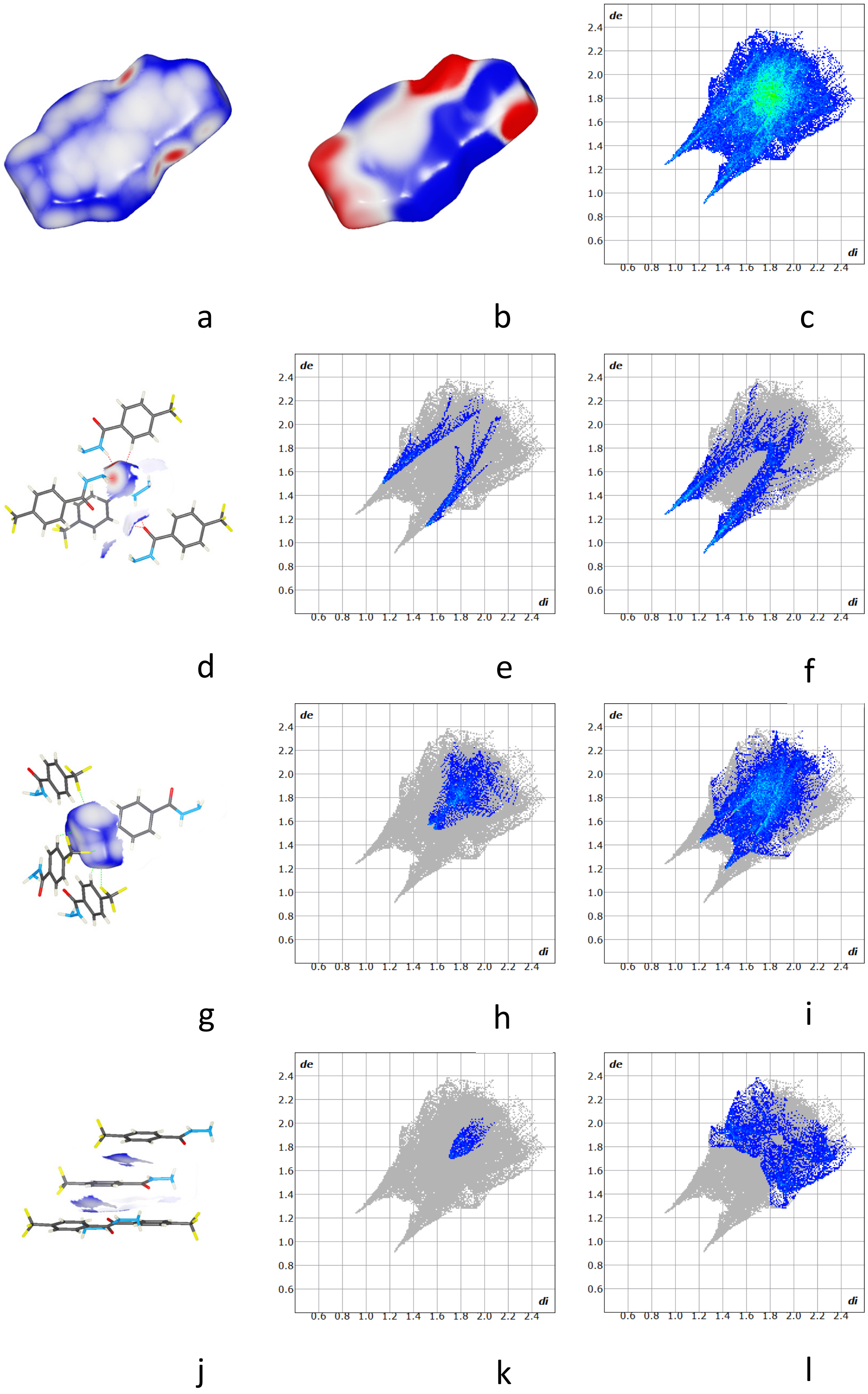

Figure S13. Hirshfeld surfaces of **1** mapped with dnorm (**a**), eletrostatic potential(**b**). Hirshfeld surface contacts for O...H(**d**), F...All(**g**) and C...All(**j**) in compound 1. (**b**) Full 2D-fingerprint plot for compound 1(**c**), and the decomposed contacts representing N...H/H...N (**e**), O...H/H...O (**f**), F...F (**h**), F...H/H...F(**i**), C...C (**k**), and C...H/H...C (**l**) intermolecular interactions.

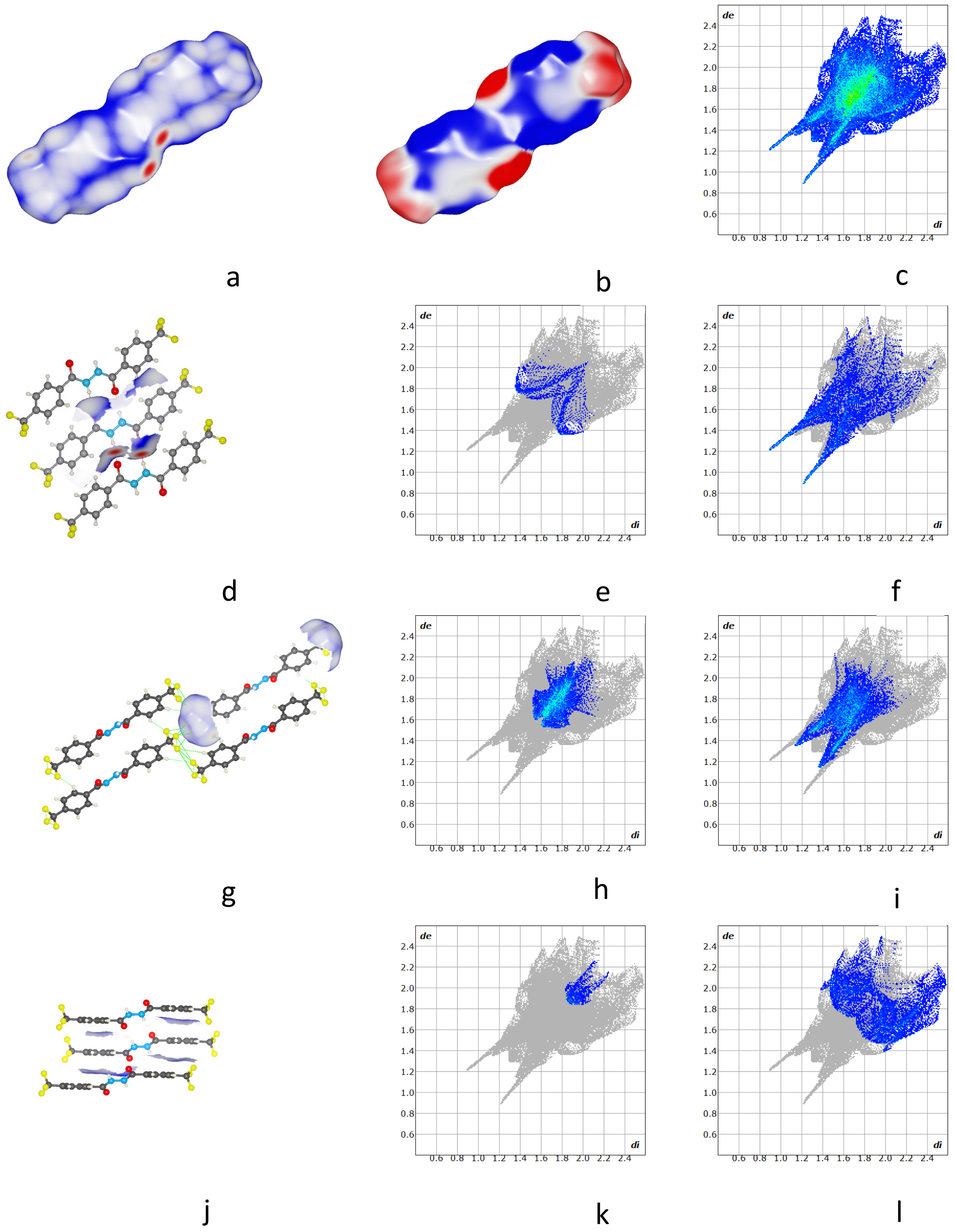

Figure S14. Hirshfeld surfaces of **2** mapped with dnorm (**a**), eletrostatic potential(**b**). Hirshfeld surface contacts for O...H(**d**), F...All(**g**) and C...All(**j**) in compound 1. (**b**) Full 2D-fingerprint plot for compound 1(**c**), and the decomposed contacts representing N...H/H...N (**e**), O...H/H...O (**f**), F...F (**h**), F...H/H...F(**i**), C...C (**k**), and C...H/H...C (**l**) intermolecular interactions.

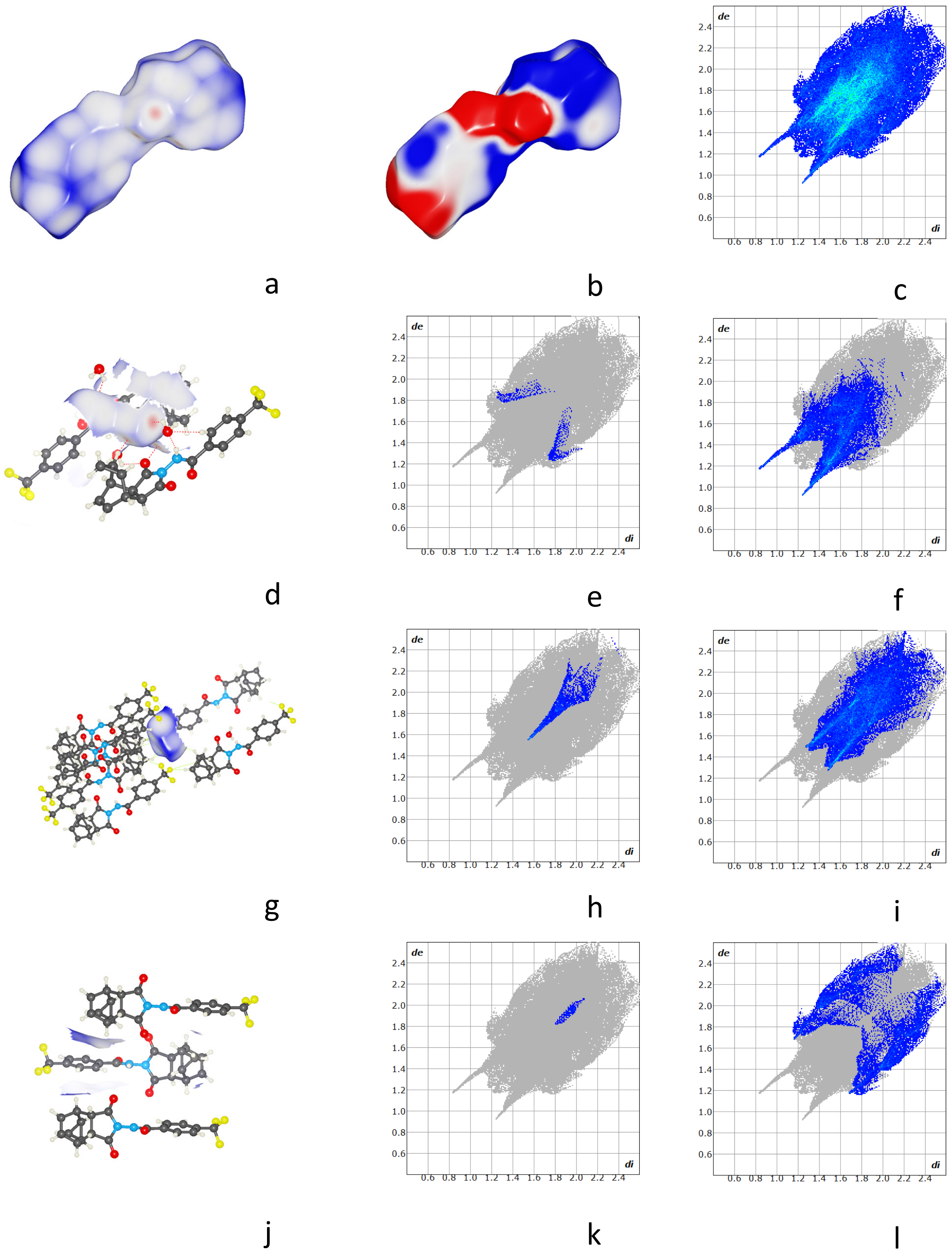

Figure S15. Hirshfeld surfaces of **3** mapped with dnorm (**a**), eletrostatic potential(**b**). Hirshfeld surface contacts for O...H(**d**), F...All(**g**) and C...All(**j**) in compound 1. (**b**) Full 2D-fingerprint plot for compound 1(**c**), and the decomposed contacts representing N...H/H...N (**e**), O...H/H...O (**f**), F...F (**h**), F...H/H...F(**i**), C...C (**k**), and C...H/H...C (**l**) intermolecular interactions.

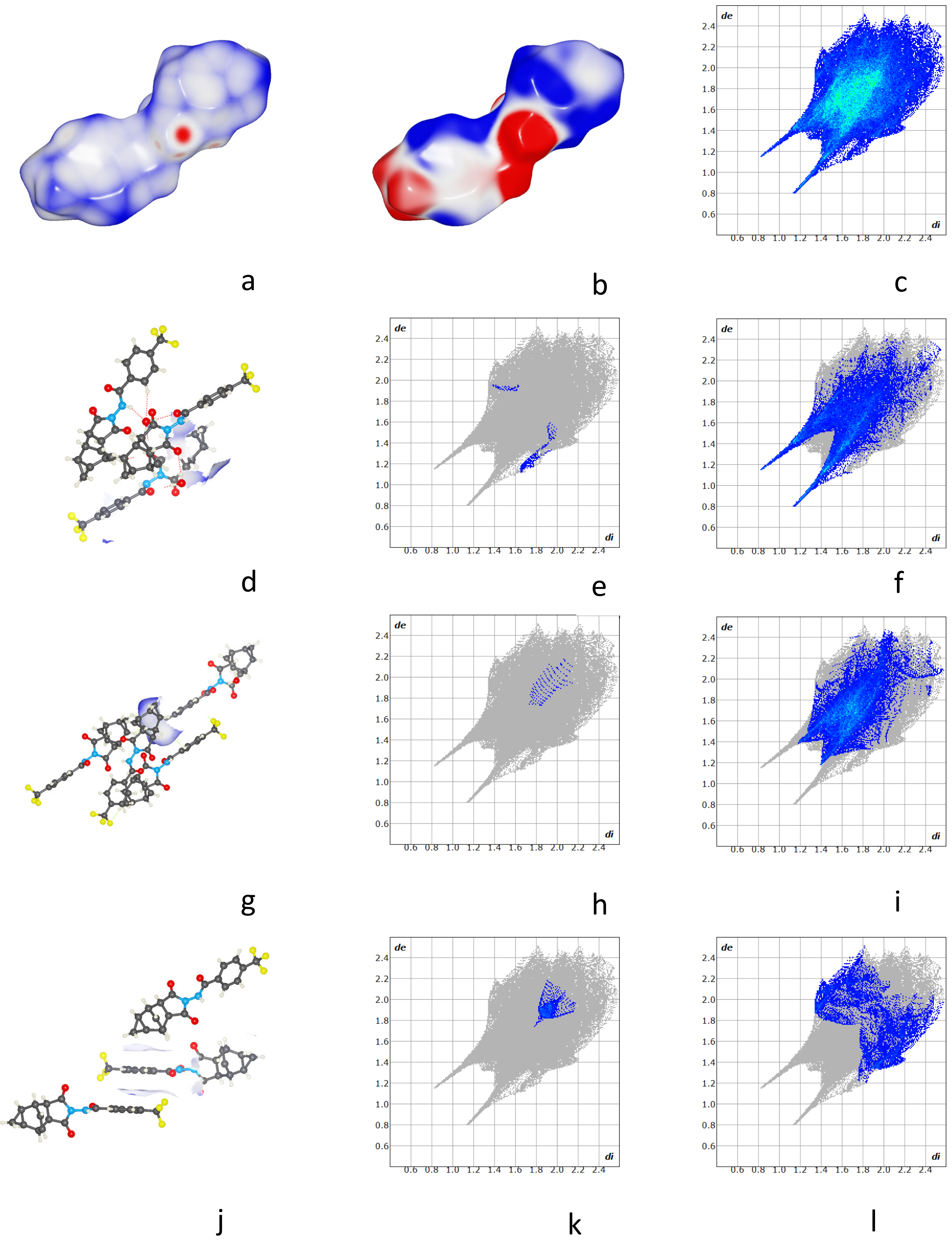

Figure S16. Hirshfeld surfaces of **4** mapped with dnorm (**a**), eletrostatic potential(**b**). Hirshfeld surface contacts for O...H(**d**), F...All(**g**) and C...All(**j**) in compound 1. (**b**) Full 2D-fingerprint plot for compound 1(**c**), and the decomposed contacts representing N...H/H...N (**e**), O...H/H...O (**f**), F...F (**h**), F...H/H...F(**i**), C...C (**k**), and C...H/H...C (**l**) intermolecular interactions.

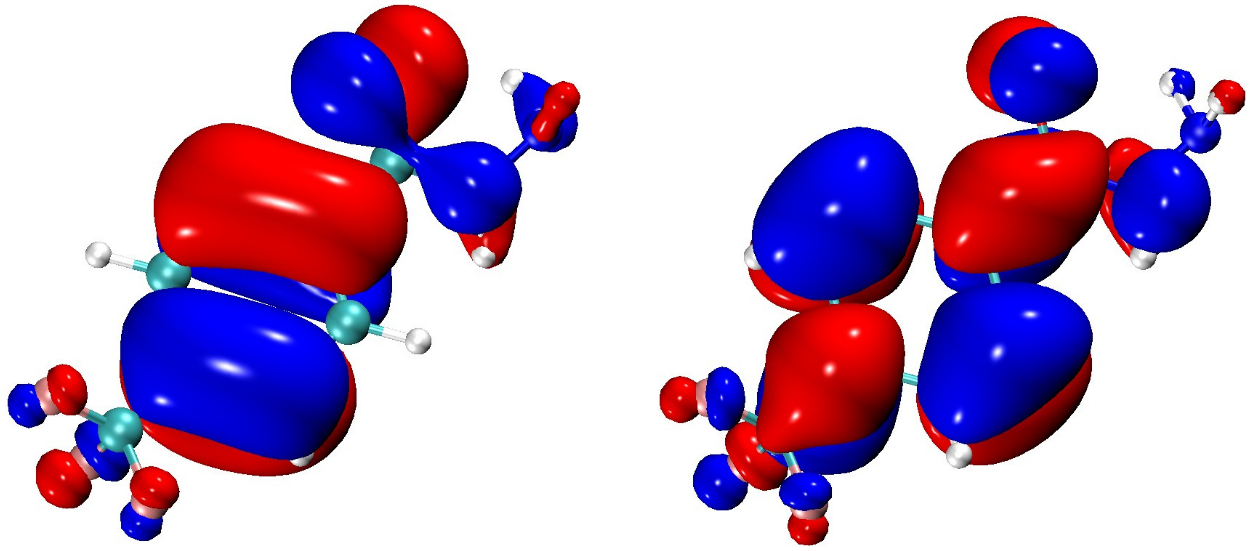

Figure S17. HOMO (left) and LUMO (right) of **1.**

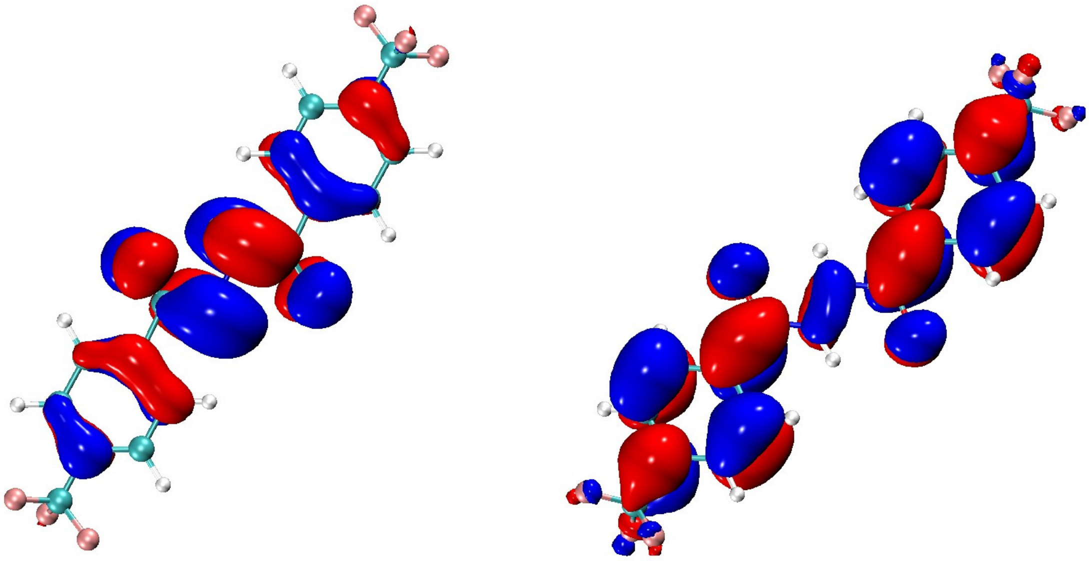

Figure S18. HOMO (left) and LUMO (right) of **2.**

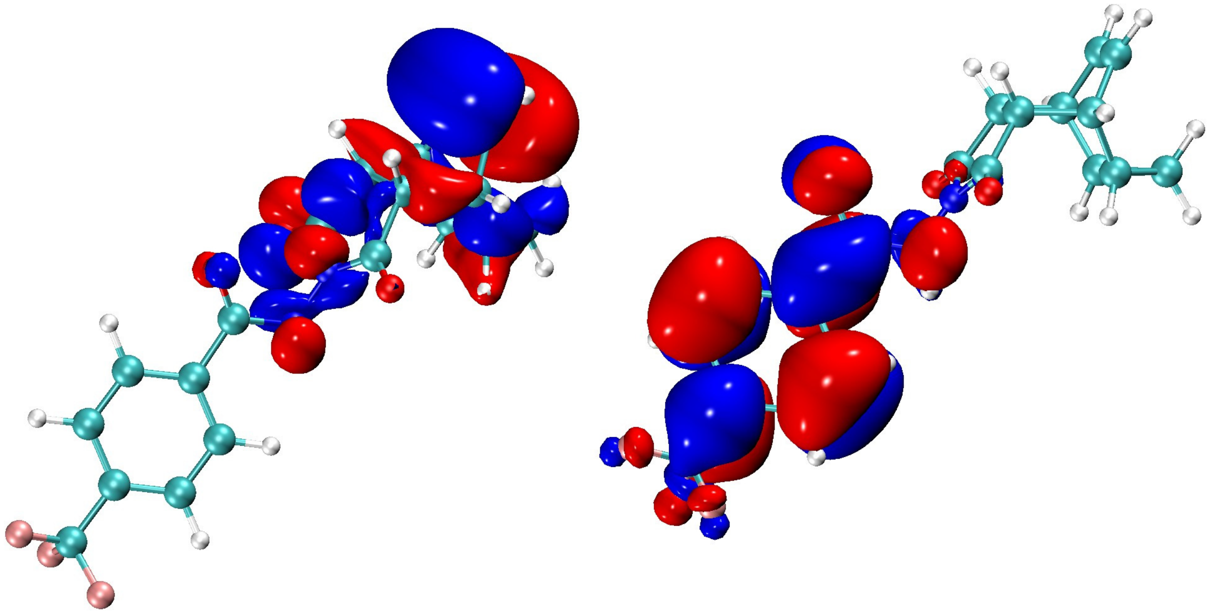

Figure S19. HOMO (left) and LUMO (right) of **3.**

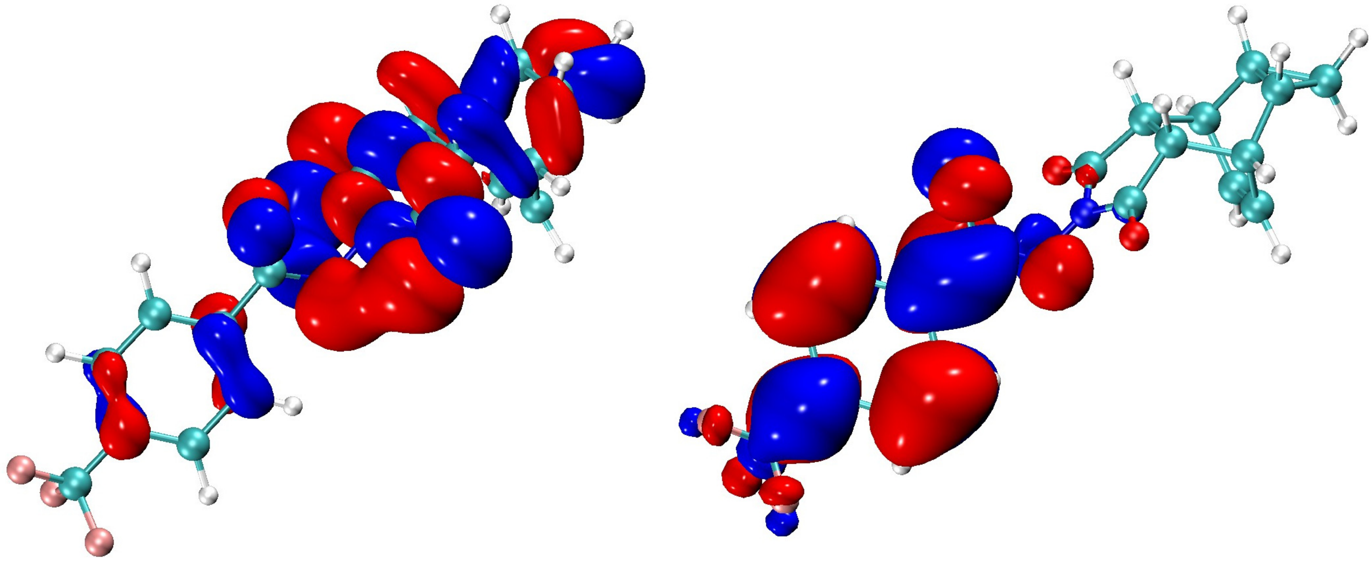

Figure S20. HOMO (left) and LUMO (right) of **4.**

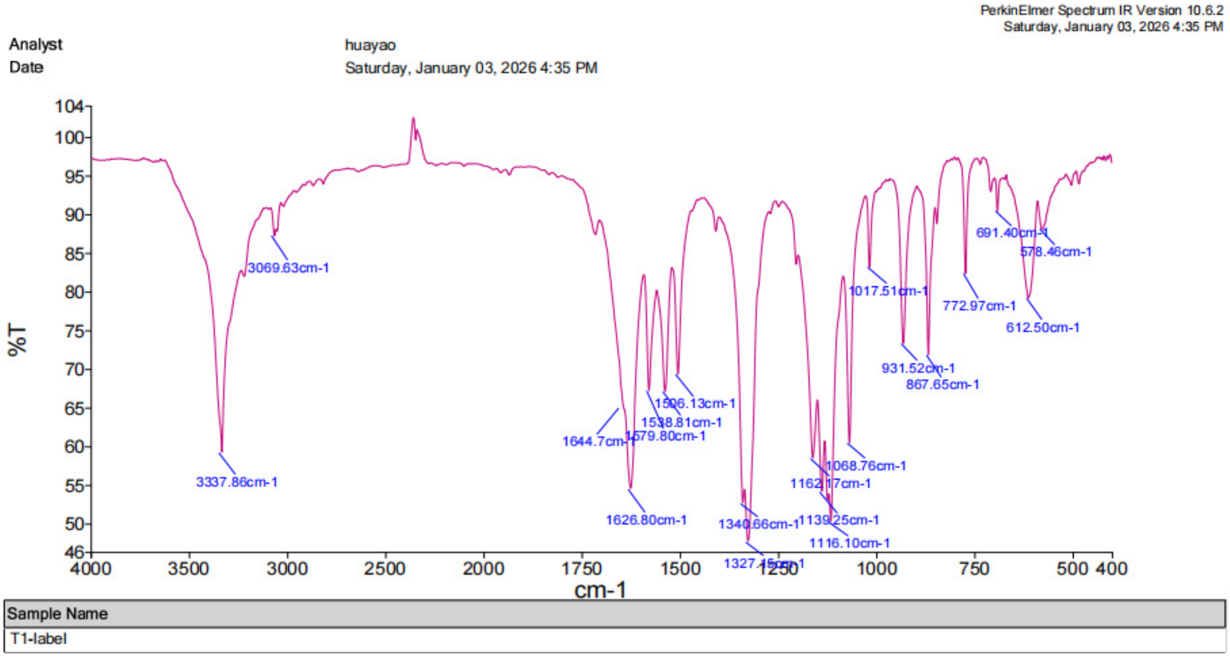

Figure S21. Original FTIR spectra of **1.**

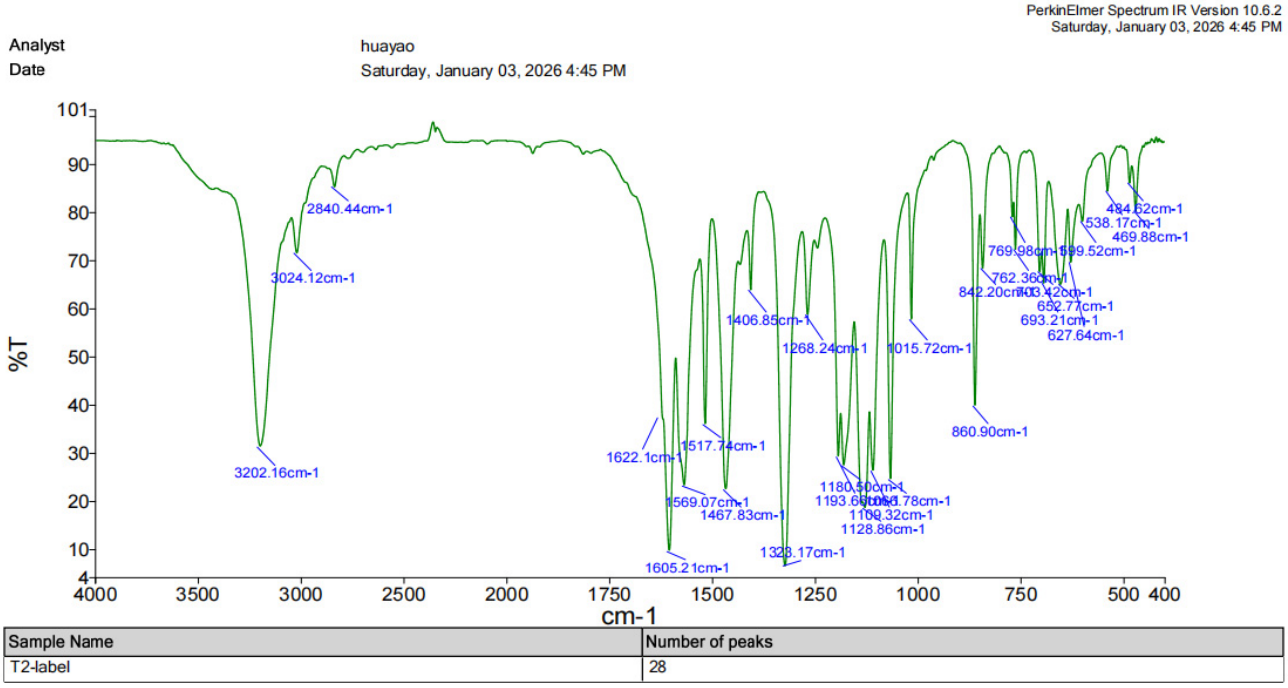

Figure S22. Original FTIR spectra of **2.**

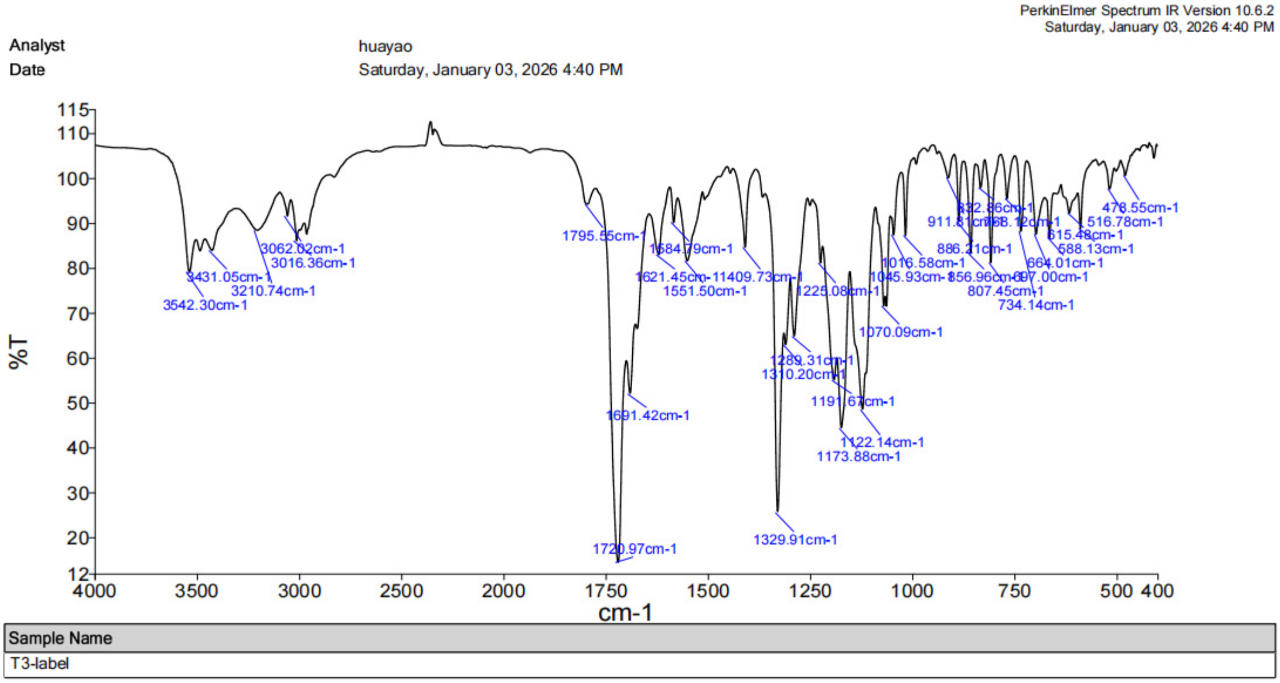

Figure S23. Original FTIR spectra of **3.**

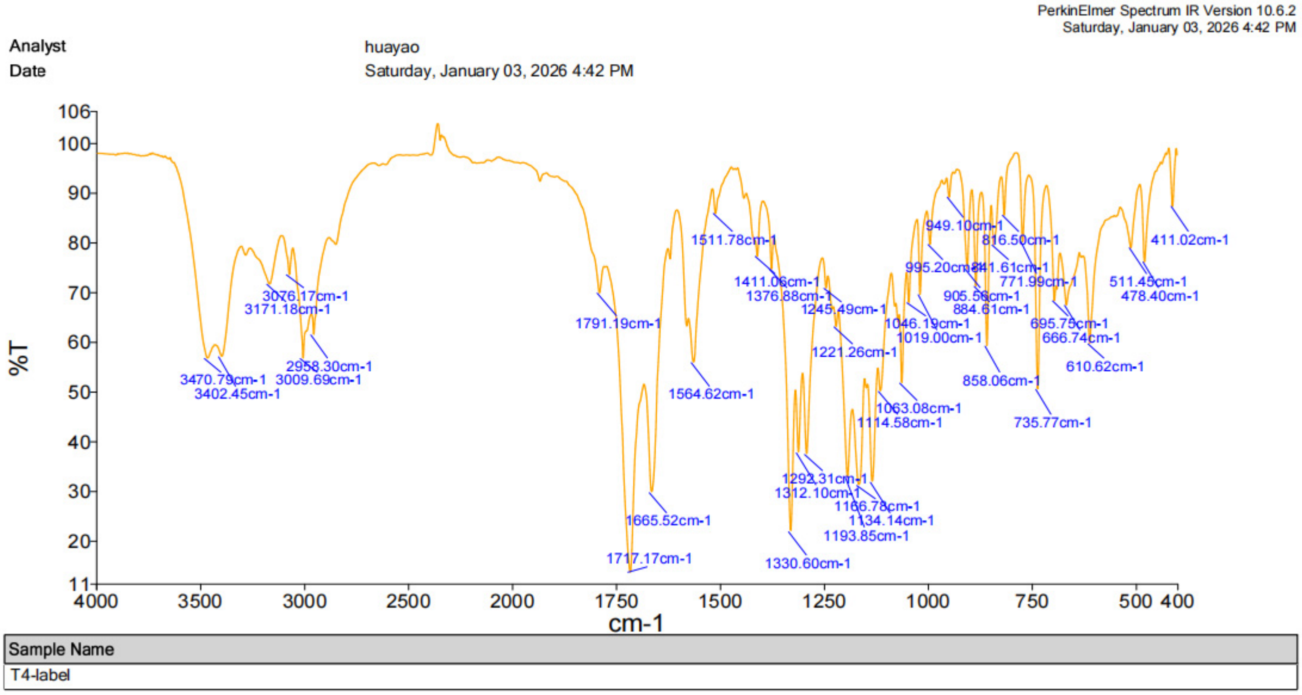

Figure S24. Original FTIR spectra of **4.**

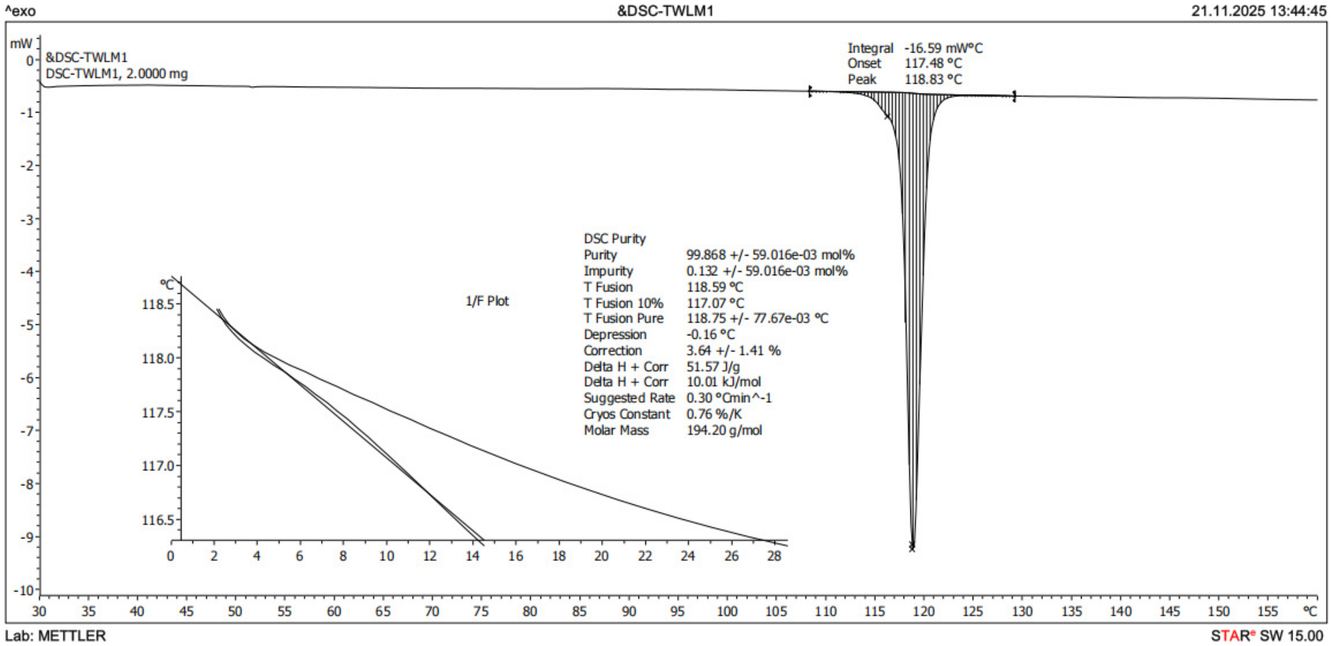

Figure S25. Original DSC profile of **1.**

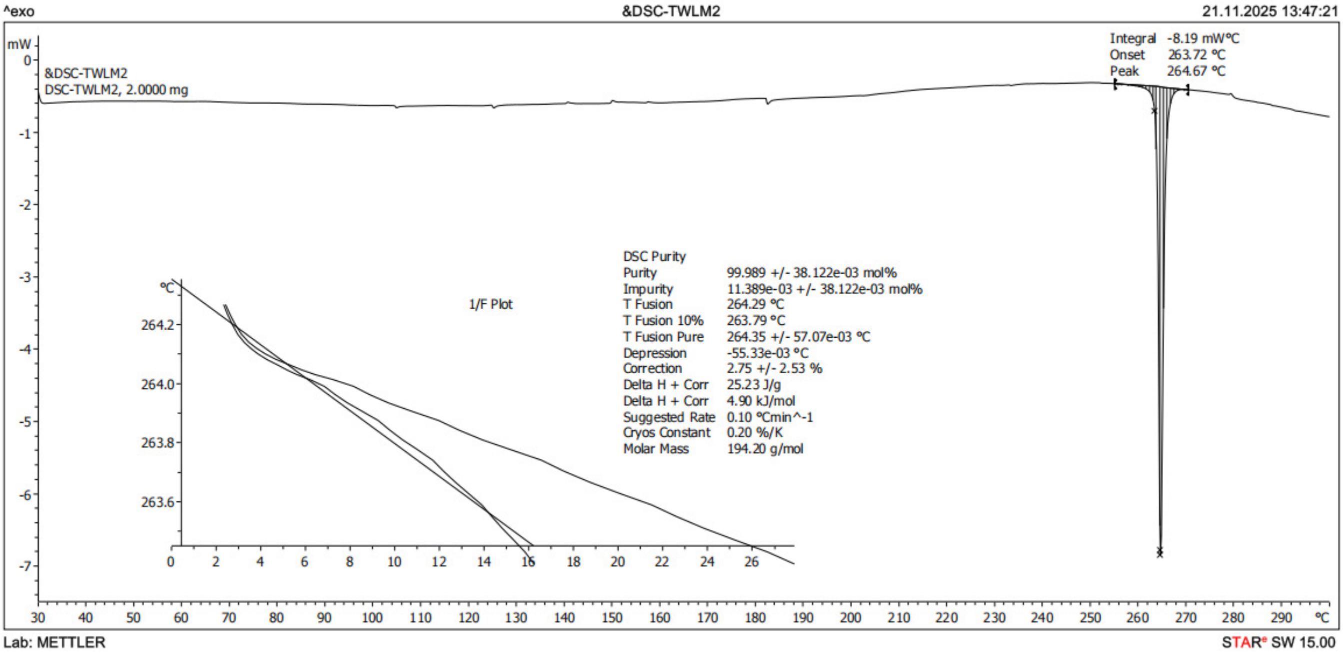

Figure S26. Original DSC profile of **2.**

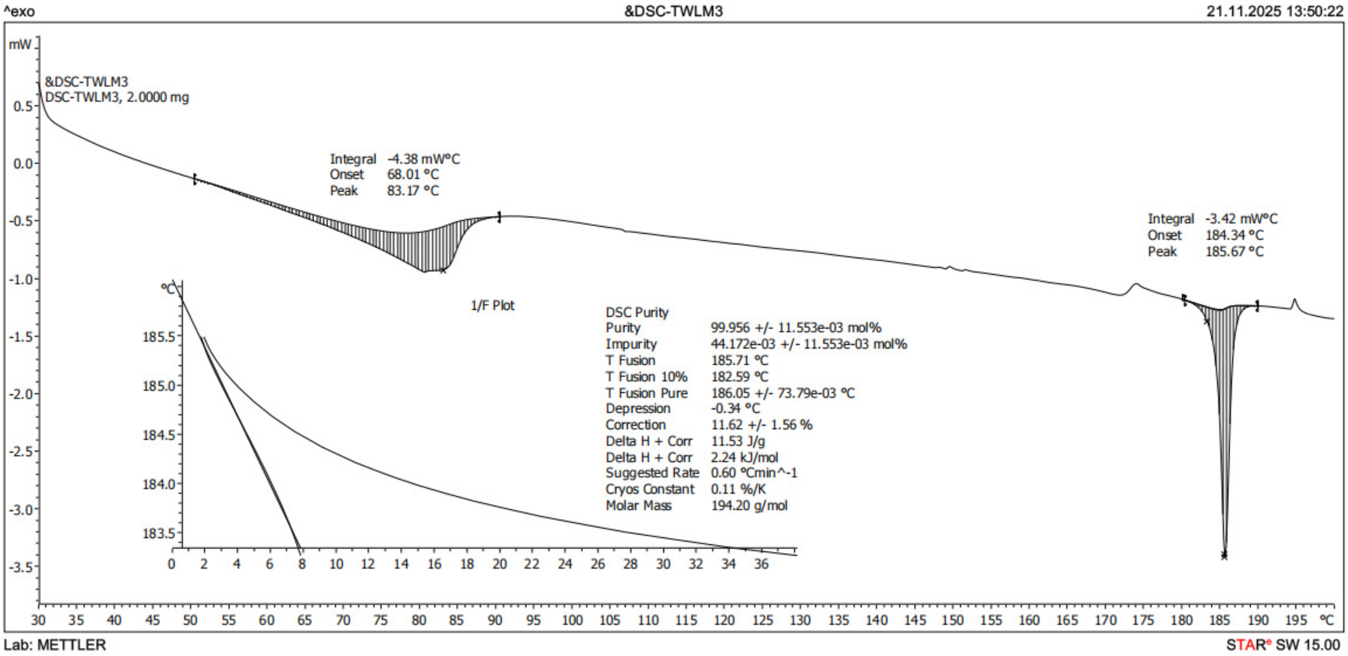

Figure S27. Original DSC profile of **3.**

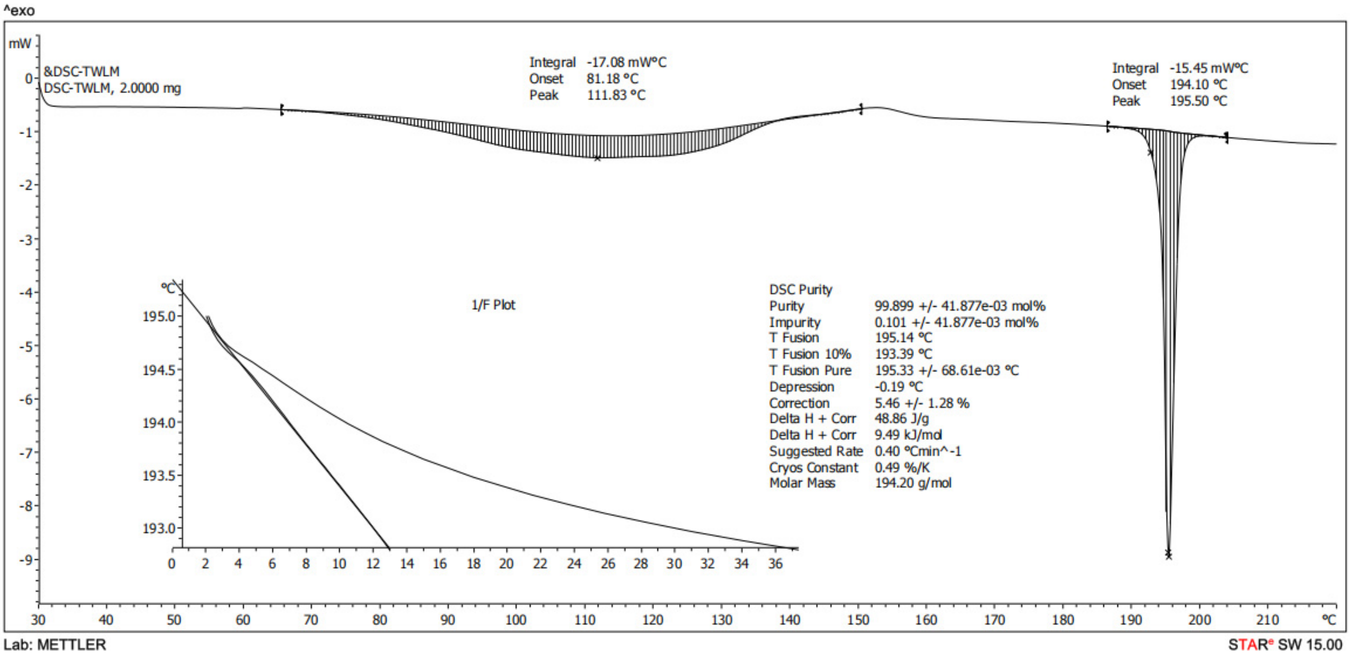

Figure S28. Original DSC profile of **4.**

Table S1. Fractional Atomic Coordinates (×10[4]) and Equivalent Isotropic Displacement Parameters (Å^2×10^3) for **1**.

Atom	*x*	*y*	*z*	U_{eq}
F1	9026(12)	-1196(10)	2128(3)	138(2)
F1A	9680(20)	-1190(20)	2152(9)	120(6)
F2	10598(3)	-2364(7)	2600(3)	116.2(19)
F2A	10450(20)	-2790(20)	2768(9)	159(7)
F3	8612(7)	-3124(6)	2690(4)	110.2(18)
F3A	8377(17)	-2730(30)	2520(13)	138(8)
O1	7334.2(10)	1890.5(13)	5232.8(6)	59.3(3)
N1	9391.2(13)	2920.3(15)	5136.6(7)	53.6(4)
N2	9223.2(15)	3914.9(18)	5676.5(9)	64.4(4)
C1	9161.6(16)	-928.2(17)	3279.4(8)	54.2(4)
C2	10238.7(16)	-170.4(18)	3562.2(8)	57.8(4)
C3	10033.1(14)	762.8(17)	4101.3(8)	53.6(4)
C4	8746.3(14)	951.8(15)	4363.1(7)	45.1(4)
C5	7672.1(15)	175.9(19)	4077.6(8)	56.6(4)
C6	7879.6(17)	-756.1(19)	3539.0(9)	60.4(4)
C7	9364(2)	-1901(2)	2681.9(10)	71.8(5)
C8	8434.4(13)	1953.4(16)	4944.5(8)	46.2(4)

Table S2. Anisotropic Displacement Parameters (Å^2×10^3) for **1**.

Atom	U_{11}	U_{22}	U_{33}	U_{23}	U_{13}	U_{12}
F1	228(7)	126(3)	60(2)	-7(2)	-21(3)	19(5)
F1A	181(13)	102(6)	75(7)	-7(6)	60(9)	-19(10)
F2	72.3(18)	155(4)	122(3)	-61(3)	33.6(19)	-7(2)
F2A	224(16)	136(7)	117(8)	-57(6)	-43(8)	128(10)
F3	134(5)	77.6(16)	119(3)	-38.6(15)	52(3)	-37.6(19)
F3A	66(4)	181(17)	166(14)	-108(12)	15(6)	-43(8)
O1	36.1(5)	65.0(7)	76.9(7)	-9.2(5)	7.1(5)	-2.8(4)
N1	39.6(6)	50.5(7)	70.8(8)	-6.3(6)	4.1(6)	-3.8(5)
N2	50.4(8)	61.0(8)	82.0(10)	-15.3(7)	-1.2(7)	1.2(6)
C1	53.5(9)	52.2(8)	57.0(9)	3.9(6)	3.9(7)	-1.7(6)
C2	42.2(7)	64.5(9)	66.7(9)	-1.5(8)	8.7(7)	-1.2(7)
C3	35.7(7)	57.6(8)	67.4(9)	0.5(7)	0.7(7)	-4.0(6)
C4	36.4(7)	42.9(7)	55.9(8)	6.8(6)	-1.6(6)	0.1(5)
C5	36.7(7)	62.3(9)	70.8(9)	-4.9(8)	3.2(6)	-5.2(6)
C6	46.1(8)	65.5(9)	69.7(10)	-7.1(8)	-2.6(7)	-9.5(7)
C7	72.0(12)	74.7(12)	68.6(11)	-8.2(9)	10.3(9)	-8.7(9)
C8	33.8(6)	43.7(7)	61.0(8)	5.1(6)	-1.5(6)	2.0(5)

Table S3. Bond Lengths for **1**.

Atom	Atom	Length/Å	Atom	Atom	Length/Å
F1	C7	1.319(7)	C1	C2	1.381(2)
F1A	C7	1.276(16)	C1	C6	1.378(2)
F2	C7	1.297(4)	C1	C7	1.491(2)
F2A	C7	1.340(11)	C2	C3	1.378(2)
F3	C7	1.314(5)	C3	C4	1.386(2)

Atom	Atom	Length/Å	Atom	Atom	Length/Å
F3A	C7	1.266(13)	C4	C5	1.389(2)
O1	C8	1.2331(17)	C4	C8	1.499(2)
N1	N2	1.407(2)	C5	C6	1.376(2)
N1	C8	1.3329(19)			

Table S4. Bond Angles for **1**.

Atom	Atom	Atom	Angle/°	Atom	Atom	Atom	Angle/°
C8	N1	N2	122.82(13)	F1A	C7	C1	114.8(9)
C2	C1	C7	120.58(15)	F2	C7	F1	106.3(4)
C6	C1	C2	119.93(15)	F2	C7	F3	105.8(4)
C6	C1	C7	119.46(15)	F2	C7	C1	114.3(3)
C3	C2	C1	120.11(14)	F2A	C7	C1	110.1(6)
C2	C3	C4	120.40(14)	F3	C7	F1	105.0(5)
C3	C4	C5	118.99(14)	F3	C7	C1	113.0(3)
C3	C4	C8	123.76(13)	F3A	C7	F1A	105.3(13)
C5	C4	C8	117.25(13)	F3A	C7	F2A	107.7(11)
C6	C5	C4	120.52(14)	F3A	C7	C1	116.1(9)
C5	C6	C1	120.04(14)	O1	C8	N1	121.22(14)
F1	C7	C1	111.8(4)	O1	C8	C4	121.37(13)
F1A	C7	F2A	101.7(10)	N1	C8	C4	117.41(12)

Table S5. Hydrogen Bonds for **1**.

D	H	A	d(D-H)/Å	d(H-A)/Å	d(D-A)/Å	D-H-A/°
N1	H1	O1[1]	0.860(16)	2.173(17)	3.0058(16)	163(2)
N2	H2A	O1[2]	0.875(16)	2.323(17)	3.180(2)	166(2)
N2	H2B	F2A[3]	0.871(16)	2.63(3)	3.30(2)	134(2)
C3	H3	O1[1]	0.93	2.45	3.3589(18)	166.7
C5	H5	N2[4]	0.93	2.65	3.536(2)	159.2
C6	H6	F1A[5]	0.93	2.64	3.473(17)	148.7
C6	H6	F2[5]	0.93	2.64	3.514(5)	157.7

[1]1/2+X,1/2-Y,1-Z; [2]3/2-X,1/2+Y,+Z; [3]2-X,-Y,1-Z; [4]-1/2+X,1/2-Y,1-Z; [5]-1/2+X,+Y,1/2-Z

Table S6. Torsion Angles for **1**.

A	B	C	D	Angle/°	A	B	C	D	Angle/°
N2	N1	C8	O1	0.5(2)	C3	C4	C8	N1	13.0(2)
N2	N1	C8	C4	-179.75(14)	C4	C5	C6	C1	0.1(3)
C1	C2	C3	C4	-0.2(2)	C5	C4	C8	O1	12.7(2)
C2	C1	C6	C5	0.3(3)	C5	C4	C8	N1	-167.05(14)
C2	C1	C7	F1	-98.9(6)	C6	C1	C2	C3	-0.3(2)
C2	C1	C7	F1A	-67.5(13)	C6	C1	C7	F1	79.4(6)
C2	C1	C7	F2	22.0(4)	C6	C1	C7	F1A	110.8(12)
C2	C1	C7	F2A	46.5(13)	C6	C1	C7	F2	-159.8(4)
C2	C1	C7	F3	143.0(4)	C6	C1	C7	F2A	-135.2(13)
C2	C1	C7	F3A	169.2(17)	C6	C1	C7	F3	-38.7(4)
C2	C3	C4	C5	0.6(2)	C6	C1	C7	F3A	-12.5(17)
C2	C3	C4	C8	-179.52(14)	C7	C1	C2	C3	177.95(16)

A	B	C	D	Angle/°	A	B	C	D	Angle/°
C3	C4	C5	C6	-0.6(2)	C7	C1	C6	C5	-177.95(16)
C3	C4	C8	O1	-167.21(14)	C8	C4	C5	C6	179.52(14)

Table S7. Hydrogen Atom Coordinates (Å×10^4) and Isotropic Displacement Parameters (Å^2×10^3) for **1**

Atom	x	y	z	U_{eq}
H1	10176(18)	2900(30)	4951(11)	80
H2A	8670(20)	4640(20)	5559(13)	97
H2B	8850(30)	3400(30)	5997(11)	97
H2	11104.49	-290.05	3388.39	69
H3	10762.18	1269.08	4290.74	64
H5	6805.6	286.96	4251.56	68
H6	7154.28	-1270.1	3350.2	72

Table S8. Atomic Occupancy for **1**.

Atom	Occupancy	Atom	Occupancy	Atom	Occupancy
F1	0.733(16)	F1A	0.267(16)	F2	0.733(16)
F2A	0.267(16)	F3	0.733(16)	F3A	0.267(16)

Table S9. Fractional Atomic Coordinates (×10^4) and Equivalent Isotropic Displacement Parameters (Å^2×10^3) for **2**.

Atom	*x*	*y*	*z*	U_{eq}
F1	-1356(6)	8479(7)	7224.6(9)	92.4(11)
F2	-3867(6)	5074(6)	7162.3(10)	90.5(10)
F3	-4794(6)	8788(7)	6835.7(9)	91.0(10)
O1	4184(7)	1174(5)	5447.5(10)	71.6(10)
N1	4158(6)	5413(6)	5168.2(10)	46.5(7)
C1	-1319(7)	6192(7)	6542.2(12)	46.6(8)
C2	-1642(7)	7410(8)	6133.4(13)	52.0(9)
C3	-105(7)	6589(8)	5778.1(12)	48.4(9)
C4	1707(7)	4549(7)	5833.9(11)	43.9(8)
C5	1972(8)	3306(8)	6243.0(13)	57.3(10)
C6	488(8)	4137(8)	6597.8(12)	54.5(10)
C7	-2831(8)	7111(8)	6937.9(13)	54.9(10)
C8	3428(7)	3560(7)	5469.1(12)	46.6(8)

Table S10. Anisotropic Displacement Parameters (Å^2×10^3) for **2**.

Atom	U_{11}	U_{22}	U_{33}	U_{23}	U_{13}	U_{12}
F1	95(2)	116(3)	66.1(17)	-33.7(16)	13.8(14)	-6.7(18)
F2	104(2)	78.5(19)	89(2)	11.1(15)	52.7(17)	0.1(17)
F3	92(2)	105(2)	75.4(18)	6.1(15)	26.9(14)	48.7(18)
O1	111(3)	33.3(14)	70.7(18)	-0.3(12)	39.5(17)	8.4(14)
N1	58.3(17)	31.5(14)	49.7(16)	-2.1(12)	18.7(13)	1.6(13)
C1	48.3(18)	43.3(19)	48.2(19)	-3.8(14)	9.6(14)	-4.5(15)
C2	45.5(19)	55(2)	55(2)	4.0(17)	9.1(15)	10.8(17)
C3	49.4(19)	50(2)	46.1(19)	5.6(15)	6.9(14)	3.7(16)
C4	48.5(18)	34.7(17)	48.6(19)	-2.1(14)	11.2(14)	-3.0(14)

Atom	U_{11}	U_{22}	U_{33}	U_{23}	U_{13}	U_{12}
C5	68(2)	48(2)	55(2)	6.9(17)	15.8(18)	15.2(19)
C6	66(2)	52(2)	45.5(19)	9.2(16)	11.5(16)	10.8(18)
C7	59(2)	53(2)	52(2)	0.3(17)	13.6(17)	7.1(18)
C8	58(2)	31.4(17)	50.8(19)	-2.9(14)	13.2(15)	-2.4(15)

Table S11. Bond Lengths for **2**.

Atom	Atom	Length/Å	Atom	Atom	Length/Å
F1	C7	1.330(5)	C1	C6	1.380(5)
F2	C7	1.317(5)	C1	C7	1.493(5)
F3	C7	1.337(5)	C2	C3	1.391(5)
O1	C8	1.233(4)	C3	C4	1.376(5)
N1	N1[1]	1.394(5)	C4	C5	1.381(5)
N1	C8	1.336(4)	C4	C8	1.493(5)
C1	C2	1.379(5)	C5	C6	1.376(5)

[1]1-X,1-Y,1-Z

Table S12. Bond Angles for **2**.

Atom	Atom	Atom	Angle/°	Atom	Atom	Atom	Angle/°
C8	N1	N1[1]	118.1(3)	C5	C6	C1	119.7(3)
C2	C1	C6	120.3(3)	F1	C7	F3	105.8(3)
C2	C1	C7	121.4(3)	F1	C7	C1	111.9(3)
C6	C1	C7	118.3(3)	F2	C7	F1	106.2(3)
C1	C2	C3	119.6(3)	F2	C7	F3	106.0(3)
C4	C3	C2	120.1(3)	F2	C7	C1	113.1(3)
C3	C4	C5	119.7(3)	F3	C7	C1	113.2(3)
C3	C4	C8	123.2(3)	O1	C8	N1	121.2(3)
C5	C4	C8	117.1(3)	O1	C8	C4	122.2(3)
C6	C5	C4	120.6(4)	N1	C8	C4	116.5(3)

[1]1-X,1-Y,1-Z

Table S13. Hydrogen Bonds for **2**.

D	H	A	d(D-H)/Å	d(H-A)/Å	d(D-A)/Å	D-H-A/°
N1	H1	O1[1]	0.85(2)	2.12(2)	2.942(4)	162(4)
C5	H5	F3[2]	0.93	2.50	3.293(5)	143.9
C6	H6	F1[3]	0.93	2.80	3.483(5)	131.1

[1]+X,1+Y,+Z; [2]1+X,-1+Y,+Z; [3]+X,-1+Y,+Z

Table S14. Torsion Angles for **2**.

A	B	C	D	Angle/°	A	B	C	D	Angle/°
N1[1]	N1	C8	O1	-1.1(7)	C3	C4	C8	N1	32.8(5)
N1[1]	N1	C8	C4	177.7(4)	C4	C5	C6	C1	1.3(6)
C1	C2	C3	C4	0.7(6)	C5	C4	C8	O1	31.1(6)
C2	C1	C6	C5	0.0(6)	C5	C4	C8	N1	-147.7(4)
C2	C1	C7	F1	-107.4(4)	C6	C1	C2	C3	-1.1(6)
C2	C1	C7	F2	132.7(4)	C6	C1	C7	F1	70.5(5)
C2	C1	C7	F3	12.0(6)	C6	C1	C7	F2	-49.4(5)

A	B	C	D	Angle/°	A	B	C	D	Angle/°
C2	C3	C4	C5	0.6(6)	C6	C1	C7	F3	-170.1(4)
C2	C3	C4	C8	-179.9(3)	C7	C1	C2	C3	176.8(4)
C3	C4	C5	C6	-1.7(6)	C7	C1	C6	C5	-177.9(4)
C3	C4	C8	O1	-148.3(4)	C8	C4	C5	C6	178.8(4)

[1]1-X,1-Y,1-Z

Table S15. Hydrogen Atom Coordinates (Å×10[4]) and Isotropic Displacement Parameters (Å[2]×10[3]) for **2**

Atom	x	y	z	U_{eq}
H1	3960(90)	7130(40)	5198(15)	70
H2	-2880.55	8773.8	6095.31	62
H3	-301.4	7418.62	5502.47	58
H5	3163.94	1895.06	6278.89	69
H6	701.65	3317.53	6873.9	65

Table S16. Fractional Atomic Coordinates (×10[4]) and Equivalent Isotropic Displacement Parameters (Å[2]×10[3]) for **3**.

Atom	x	y	z	U_{eq}
F1	4566(15)	8020(20)	16(7)	210(7)
F1A	1146(12)	7250(20)	494(6)	198(8)
F2	2120(30)	6158(9)	7(8)	197(7)
F2A	3880(30)	8922(9)	450(8)	195(8)
F3	1980(20)	8382(16)	627(4)	160(5)
F3A	3479(18)	6660(12)	-212(3)	141(4)
O1	9376.8(17)	6659.0(12)	3722.0(8)	55.3(3)
O2	8968(2)	9963.0(13)	5952.0(9)	70.6(4)
O3	7384.5(17)	4808.5(11)	4996.3(7)	53.5(3)
N1	6927(2)	7404.1(15)	4503.1(8)	50.2(3)
N2	8054.6(18)	7392.8(13)	5295.9(8)	46.3(3)
C1	4404(4)	7435(2)	1362.2(12)	74.3(5)
C2	3807(4)	7976(2)	2166.2(13)	73.9(5)
C3	4881(3)	7892(2)	2946.6(12)	65.5(5)
C4	6542(2)	7238.0(16)	2928.7(10)	47.6(3)
C5	7140(4)	6697(3)	2116.7(13)	79.7(6)
C6	6075(4)	6802(3)	1336.7(14)	99.0(8)
C8	7768(2)	7080.8(15)	3744.8(10)	44.8(3)
C7	3215(6)	7547(4)	529.6(16)	114.7(11)
C9	9038(2)	8721.0(16)	5983.5(11)	49.5(3)
C10	10076(2)	8302.4(16)	6746.2(10)	47.8(3)
C11	9496(2)	6557.1(16)	6431.4(9)	44.3(3)
C12	8197(2)	6074.0(15)	5498.2(9)	43.4(3)
C13	9220(3)	8861.7(19)	7626.0(11)	57.0(4)
C14	10118(3)	8167(2)	8281.8(11)	66.5(5)
C15	9596(3)	6663(2)	8004.9(12)	64.2(4)
C16	8214(3)	5942.9(18)	7092.6(10)	52.9(4)
C17	6219(2)	6509(2)	7062.0(11)	57.0(4)
C18	6804(3)	8208(2)	7375.2(12)	60.2(4)

Atom	*x*	*y*	*z*	U_{eq}
C19	5575(3)	7324(3)	7895.7(14)	80.1(6)
O4	3353.2(19)	8405.2(15)	5039.4(9)	64.9(3)

Table S17. Anisotropic Displacement Parameters (Å^2×10^3) for **3**.

Atom	U_{11}	U_{22}	U_{33}	U_{23}	U_{13}	U_{12}
F1	306(10)	320(20)	105(6)	134(10)	71(7)	177(13)
F1A	128(4)	370(20)	117(6)	88(10)	-19(3)	103(8)
F2	261(14)	194(7)	102(7)	0(5)	-83(8)	104(7)
F2A	318(17)	144(5)	130(7)	84(5)	-47(8)	67(6)
F3	244(11)	224(9)	66(2)	40(5)	3(5)	184(9)
F3A	209(8)	180(8)	43.4(17)	21(3)	-3(3)	101(6)
O1	55.9(6)	54.9(6)	63.9(7)	22.7(5)	12.1(5)	25.7(5)
O2	85.1(8)	43.0(6)	83.9(9)	27.9(6)	-0.4(6)	15.4(5)
O3	62.1(6)	44.0(6)	52.1(6)	12.1(5)	5.2(5)	15.2(4)
N1	52.3(6)	61.1(8)	46.6(7)	24.7(6)	7.2(5)	25.1(6)
N2	51.4(6)	46.4(6)	45.0(6)	18.8(5)	3.7(5)	17.6(5)
C1	101.9(14)	77.0(12)	50.1(10)	21.2(9)	1.8(9)	39.5(11)
C2	100.0(14)	80.0(12)	57.3(10)	23.5(9)	6.9(9)	54.6(11)
C3	90.8(12)	74.2(11)	48.0(9)	22.3(8)	14.1(8)	49.3(10)
C4	56.8(8)	41.3(7)	47.9(8)	17.4(6)	8.5(6)	15.3(6)
C5	90.8(13)	109.8(16)	56.1(10)	25.4(10)	19.0(9)	58.3(12)
C6	127.2(19)	141(2)	47.7(10)	24.2(12)	21.1(11)	75.6(18)
C8	49.5(7)	37.2(6)	50.6(8)	17.4(6)	7.6(6)	13.5(5)
C7	163(3)	141(3)	53.0(13)	24.2(16)	-0.5(16)	82(2)
C9	49.1(7)	43.8(7)	58.1(9)	20.1(6)	7.4(6)	13.2(6)
C10	43.5(7)	46.2(7)	52.3(8)	15.8(6)	1.8(6)	11.9(6)
C11	46.8(7)	46.8(7)	44.7(7)	17.2(6)	7.5(5)	20.0(6)
C12	46.4(7)	44.0(7)	46.5(7)	18.3(6)	11.3(6)	18.3(5)
C13	60.1(9)	52.2(8)	52.6(9)	5.6(7)	1.1(7)	20.3(7)
C14	63.9(10)	88.6(13)	43.7(8)	12.6(8)	-1.0(7)	28.6(9)
C15	71.8(10)	85.2(13)	51.3(9)	32.4(9)	11.2(7)	38.1(9)
C16	64.7(9)	51.5(8)	50.4(8)	23.8(7)	13.0(7)	20.3(7)
C17	50.3(8)	66.9(10)	54.8(9)	22.1(7)	10.1(6)	13.7(7)
C18	57.1(8)	69.0(10)	58.4(9)	14.5(8)	9.9(7)	30.8(8)
C19	63.7(10)	116.8(17)	70.9(12)	30.4(12)	26.3(9)	36.9(11)
O4	59.2(7)	61.7(7)	76.3(8)	23.1(6)	11.2(6)	19.7(5)

Table S18. Bond Lengths for **3**.

Atom	Atom	Length/Å	Atom	Atom	Length/Å
F1	C7	1.345(7)	C3	C4	1.375(2)
F1A	C7	1.315(8)	C4	C5	1.376(2)
F2	C7	1.335(7)	C4	C8	1.498(2)
F2A	C7	1.325(6)	C5	C6	1.382(3)
F3	C7	1.253(5)	C9	C10	1.503(2)
F3A	C7	1.296(5)	C10	C11	1.540(2)
O1	C8	1.2155(17)	C10	C13	1.555(2)

Atom	Atom	Length/Å	Atom	Atom	Length/Å
O2	C9	1.2070(18)	C11	C12	1.506(2)
O3	C12	1.2062(17)	C11	C16	1.556(2)
N1	N2	1.3809(16)	C13	C14	1.503(2)
N1	C8	1.3574(19)	C13	C18	1.530(2)
N2	C9	1.383(2)	C14	C15	1.326(3)
N2	C12	1.3952(17)	C15	C16	1.498(2)
C1	C2	1.365(3)	C16	C17	1.529(2)
C1	C6	1.369(3)	C17	C18	1.499(2)
C1	C7	1.490(3)	C17	C19	1.490(3)
C2	C3	1.376(2)	C18	C19	1.493(3)

Table S19. Bond Angles for **3**.

Atom	Atom	Atom	Angle/°	Atom	Atom	Atom	Angle/°
C8	N1	N2	118.47(12)	F3A	C7	C1	114.5(4)
N1	N2	C9	121.47(12)	O2	C9	N2	123.80(14)
N1	N2	C12	123.97(12)	O2	C9	C10	128.45(15)
C9	N2	C12	114.45(12)	N2	C9	C10	107.71(11)
C2	C1	C6	119.51(17)	C9	C10	C11	105.18(11)
C2	C1	C7	119.1(2)	C9	C10	C13	111.84(12)
C6	C1	C7	121.4(2)	C11	C10	C13	109.59(12)
C1	C2	C3	120.33(17)	C10	C11	C16	109.24(12)
C4	C3	C2	120.75(16)	C12	C11	C10	105.51(11)
C3	C4	C5	118.65(15)	C12	C11	C16	112.00(12)
C3	C4	C8	124.02(14)	O3	C12	N2	124.16(13)
C5	C4	C8	117.32(14)	O3	C12	C11	128.75(12)
C4	C5	C6	120.38(18)	N2	C12	C11	107.09(11)
C1	C6	C5	120.35(18)	C14	C13	C10	106.20(13)
O1	C8	N1	122.45(13)	C14	C13	C18	109.89(15)
O1	C8	C4	122.15(13)	C18	C13	C10	105.56(12)
N1	C8	C4	115.38(12)	C15	C14	C13	114.44(15)
F1	C7	C1	110.6(4)	C14	C15	C16	114.73(15)
F1A	C7	F2A	107.8(6)	C15	C16	C11	105.92(13)
F1A	C7	C1	113.7(5)	C15	C16	C17	110.03(13)
F2	C7	F1	102.7(6)	C17	C16	C11	106.00(12)
F2	C7	C1	109.3(4)	C18	C17	C16	110.57(13)
F2A	C7	C1	110.8(4)	C19	C17	C16	121.88(15)
F3	C7	F1	107.3(6)	C19	C17	C18	59.95(13)
F3	C7	F2	108.8(6)	C17	C18	C13	110.57(13)
F3	C7	C1	117.2(3)	C19	C18	C13	122.16(16)
F3A	C7	F1A	105.6(6)	C19	C18	C17	59.71(13)
F3A	C7	F2A	103.8(7)	C17	C19	C18	60.34(12)

Table S20. Hydrogen Bonds for **3**.

D	H	A	d(D-H)/Å	d(H-A)/Å	d(D-A)/Å	D-H-A/°
N1	H1	O4	0.868(15)	2.007(16)	2.8584(17)	166(2)
C3	H3	O4	0.93	2.60	3.482(2)	159.4

D	H	A	d(D-H)/Å	d(H-A)/Å	d(D-A)/Å	D-H-A/°
C6	H6	F2[1]	0.93	2.85	3.558(13)	134.0
C11	H11	O1[2]	0.98	2.66	3.2643(16)	120.1
C11	H11	O3[2]	0.98	2.69	3.4036(17)	130.1
C13	H13	F3[3]	0.98	2.92	3.546(6)	122.8
C15	H15	F2[4]	0.93	2.78	3.592(8)	146.4
C15	H15	F3A[4]	0.93	2.85	3.576(7)	135.6
C16	H16	O1[2]	0.98	2.68	3.2885(18)	120.7
C17	H17	O3[5]	0.98	2.56	3.520(2)	165.6
C19	H19A	F1[6]	0.97	2.78	3.385(8)	120.9
O4	H4A	O1[7]	0.849(17)	2.33(2)	2.9710(17)	132(2)
O4	H4A	O3[5]	0.849(17)	2.37(2)	2.9672(17)	128(2)
O4	H4B	O2[3]	0.847(17)	2.166(17)	3.0058(18)	171(2)

[1]1-X,1-Y,-Z; [2]2-X,1-Y,1-Z; [3]1-X,2-Y,1-Z; [4]1+X,+Y,1+Z; [5]1-X,1-Y,1-Z; [6]+X,+Y,1+Z; [7]-1+X,+Y,+Z

Table S21. Torsion Angles for **3**.

A	B	C	D	Angle/˚	A	B	C	D	Angle/˚
O2	C9	C10	C11	175.54(15)	C9	N2	C12	O3	177.58(13)
O2	C9	C10	C13	56.7(2)	C9	N2	C12	C11	-1.85(16)
N1	N2	C9	O2	1.0(2)	C9	C10	C11	C12	1.16(14)
N1	N2	C9	C10	178.93(12)	C9	C10	C11	C16	-119.41(12)
N1	N2	C12	O3	1.4(2)	C9	C10	C13	C14	171.38(13)
N1	N2	C12	C11	-178.05(12)	C9	C10	C13	C18	54.72(16)
N2	N1	C8	O1	6.4(2)	C10	C11	C12	O3	-179.10(14)
N2	N1	C8	C4	-175.25(11)	C10	C11	C12	N2	0.29(14)
N2	C9	C10	C11	-2.22(15)	C10	C11	C16	C15	-56.68(15)
N2	C9	C10	C13	-121.11(13)	C10	C11	C16	C17	60.22(15)
C1	C2	C3	C4	-1.3(3)	C10	C13	C14	C15	-59.00(19)
C2	C1	C6	C5	0.6(4)	C10	C13	C18	C17	61.48(17)
C2	C1	C7	F1	136.3(9)	C10	C13	C18	C19	127.87(17)
C2	C1	C7	F1A	-40.4(11)	C11	C10	C13	C14	55.14(16)
C2	C1	C7	F2	-111.4(10)	C11	C10	C13	C18	-61.52(15)
C2	C1	C7	F2A	81.1(11)	C11	C16	C17	C18	-61.79(16)
C2	C1	C7	F3	13.0(11)	C11	C16	C17	C19	-128.43(17)
C2	C1	C7	F3A	-161.9(7)	C12	N2	C9	O2	-175.26(14)
C2	C3	C4	C5	1.4(3)	C12	N2	C9	C10	2.63(16)
C2	C3	C4	C8	-179.00(17)	C12	C11	C16	C15	-173.20(12)
C3	C4	C5	C6	-0.5(3)	C12	C11	C16	C17	-56.30(15)
C3	C4	C8	O1	-171.27(16)	C13	C10	C11	C12	121.54(12)
C3	C4	C8	N1	10.4(2)	C13	C10	C11	C16	0.97(15)
C4	C5	C6	C1	-0.5(4)	C13	C14	C15	C16	0.1(2)
C5	C4	C8	O1	8.4(2)	C13	C18	C19	C17	-96.52(17)
C5	C4	C8	N1	-170.01(16)	C14	C13	C18	C17	-52.65(18)
C6	C1	C2	C3	0.3(4)	C14	C13	C18	C19	13.7(2)
C6	C1	C7	F1	-43.7(10)	C14	C15	C16	C11	59.38(19)
C6	C1	C7	F1A	139.5(10)	C14	C15	C16	C17	-54.8(2)
C6	C1	C7	F2	68.6(10)	C15	C16	C17	C18	52.31(18)
C6	C1	C7	F2A	-98.9(10)	C15	C16	C17	C19	-14.3(2)

A	B	C	D	Angle/°	A	B	C	D	Angle/°
C6	C1	C7	F3	-167.1(10)	C16	C11	C12	O3	-60.36(19)
C6	C1	C7	F3A	18.0(8)	C16	C11	C12	N2	119.03(12)
C8	N1	N2	C9	108.61(15)	C16	C17	C18	C13	0.28(19)
C8	N1	N2	C12	-75.44(18)	C16	C17	C18	C19	-115.76(16)
C8	C4	C5	C6	179.9(2)	C16	C17	C19	C18	96.79(17)
C7	C1	C2	C3	-179.8(2)	C18	C13	C14	C15	54.7(2)
C7	C1	C6	C5	-179.3(3)	C19	C17	C18	C13	116.05(17)

Table S22. Hydrogen Atom Coordinates (Å×10^4) and Isotropic Displacement Parameters (Å^2×10^3) for **3**.

Atom	*x*	*y*	*z*	U_{eq}
H1	5810(30)	7730(20)	4578(14)	75
H2	2668.83	8403.59	2186.7	89
H3	4478.99	8282.69	3492.3	79
H5	8266.36	6257.58	2092.71	96
H6	6497.09	6440.8	791.4	119
H10	11621.85	8720.97	6850.72	57
H11	10791.51	6215.08	6411.83	53
H13	9627.86	9971.42	7861.86	68
H14	10970.01	8743.28	8835.82	80
H15	10050.93	6100.25	8350.02	77
H16	7855.34	4833.48	6923.63	63
H17	5094.65	5992.15	6530.12	68
H18	6023.49	8697.86	7029.29	72
H19A	6308.77	7329.53	8467.97	96
H19B	4084.42	7292.51	7872.02	96
H4A	2410(30)	7560(20)	4841(17)	97
H4B	2640(40)	8890(30)	4811(16)	97

Table S23. Atomic Occupancy for **3**.

Atom	Occupancy	Atom	Occupancy	Atom	Occupancy
F1	0.493(18)	F1A	0.507(18)	F2	0.493(18)
F2A	0.507(18)	F3	0.493(18)	F3A	0.507(18)

Table S24. Fractional Atomic Coordinates (×10^4) and Equivalent Isotropic Displacement Parameters (Å^2×10^3) for **4**

Atom	*x*	*y*	*z*	U_{eq}
F1	3367.1(13)	6659(2)	5525.8(6)	118.8(8)
F2	4809.1(13)	7120.5(18)	5885.2(5)	91.1(5)
F3	3988.8(19)	8531.0(19)	5465.3(6)	122.5(8)
O1	7224.5(10)	6228.6(19)	3692.5(5)	70.7(5)
O2	6181.7(11)	7331.2(12)	2586.7(5)	51.4(3)
O3	6322.3(13)	3114.6(14)	2942.8(6)	64.8(4)
N1	5733.1(12)	5507.8(15)	3300.6(5)	40.9(3)
N2	6209.0(10)	5273.9(13)	2861.4(5)	38.1(3)
C1	4743.7(15)	6934(2)	5004.9(6)	50.1(5)
C2	4223.6(15)	6243(2)	4631.3(7)	52.8(5)
C3	4707.2(14)	5932(2)	4202.4(7)	49.6(5)

Atom	*x*	*y*	*z*	U_{eq}
C4	5714.7(13)	6298.8(18)	4149.1(6)	43.2(4)
C5	6227.2(17)	7009(3)	4527.8(8)	73.4(7)
C6	5740.6(18)	7329(3)	4952.3(8)	77.9(8)
C7	4219.9(18)	7313(3)	5463.7(7)	64.3(6)
C8	6297.7(13)	6001.1(19)	3697.1(6)	44.2(4)
C9	6400.3(12)	6228.2(16)	2525.6(6)	37.5(4)
C10	6908.7(13)	5635.4(16)	2094.4(6)	38.5(4)
C11	6923.3(13)	4197.4(17)	2204.1(6)	41.9(4)
C12	6465.4(14)	4053.3(17)	2706.3(6)	42.7(4)
C13	6287.0(15)	5903.7(17)	1584.0(6)	44.5(4)
C14	5230.8(15)	5339(2)	1621.6(7)	49.5(4)
C15	5228.5(15)	4108(2)	1715.9(7)	51.6(5)
C16	6280.2(16)	3502.5(18)	1768.9(7)	50.0(4)
C17	6881.2(18)	3778(2)	1307.4(7)	57.6(5)
C18	6891.8(17)	5184(2)	1203.4(7)	54.7(5)
C19	6361(2)	4295(2)	827.9(7)	66.6(6)
O4	3774.0(10)	4489.7(15)	3077.1(5)	56.0(4)

Table S25. Anisotropic Displacement Parameters ($Å^2 \times 10^3$) for 4

Atom	U_{11}	U_{22}	U_{33}	U_{23}	U_{13}	U_{12}
F1	81.3(10)	205(2)	76.0(10)	-56.9(12)	42.8(8)	-34.2(12)
F2	107.8(12)	129.5(14)	36.3(6)	-16.0(7)	7.8(7)	11.6(10)
F3	191(2)	106.5(14)	76.9(11)	-2.0(9)	54.9(12)	65.9(14)
O1	40.4(7)	121.3(14)	52.2(8)	-23.7(8)	15.0(6)	-15.3(8)
O2	68.7(8)	38.8(7)	48.7(7)	-5.6(5)	17.6(6)	1.4(6)
O3	92.4(11)	48.1(8)	56.8(8)	14.1(6)	24.6(8)	15.1(7)
N1	39.1(7)	53.8(9)	31.3(7)	-6.2(6)	12.5(5)	-2.5(6)
N2	41.4(7)	42.7(8)	31.6(6)	-2.9(5)	12.0(5)	2.1(6)
C1	51.9(10)	65.8(12)	33.4(8)	-6.6(8)	8.2(7)	6.7(9)
C2	42.2(9)	75.8(13)	41.6(9)	-9.5(9)	11.0(7)	-0.5(9)
C3	43.2(9)	69.8(12)	36.3(9)	-12.5(8)	7.1(7)	-4.0(8)
C4	40.6(8)	57.7(11)	32.1(8)	-4.3(7)	6.9(6)	0.4(7)
C5	48.0(11)	122(2)	51.5(11)	-30.2(13)	11.4(9)	-22.6(12)
C6	61.2(13)	125(2)	48.4(11)	-37.4(13)	8.5(10)	-21.3(14)
C7	68.6(13)	85.8(16)	39.8(10)	-14.3(10)	12.3(9)	8.5(12)
C8	38.1(8)	58.9(11)	36.7(8)	-3.9(7)	9.4(6)	-1.2(7)
C9	38.4(8)	40.4(9)	34.2(8)	-4.2(6)	6.5(6)	-2.8(6)
C10	39.2(8)	43.1(9)	34.5(8)	-5.8(6)	10.9(6)	-4.9(7)
C11	44.3(9)	44.0(9)	38.4(8)	-3.6(7)	10.6(7)	7.9(7)
C12	46.7(9)	43.7(9)	38.4(8)	1.9(7)	7.4(7)	8.8(7)
C13	59.3(10)	42.0(9)	33.1(8)	0.5(7)	8.8(7)	-5.2(8)
C14	47.7(10)	61.6(12)	39.1(9)	1.3(8)	2.0(7)	2.1(8)
C15	51.7(10)	61.9(12)	41.7(9)	-3.3(8)	7.7(7)	-17.2(9)
C16	69.8(12)	38.0(9)	43.6(9)	-6.8(7)	13.1(8)	-4.2(8)
C17	71.5(13)	60.2(12)	43.4(10)	-14.8(9)	18.4(9)	1.3(10)
C18	66.3(12)	64.5(12)	35.4(9)	-6.2(8)	18.2(8)	-14.9(10)

Atom	U_{11}	U_{22}	U_{33}	U_{23}	U_{13}	U_{12}
C19	88.6(16)	75.7(15)	36.9(10)	-15.2(10)	14.5(10)	-12.5(12)
O4	44.5(7)	68.5(9)	56.6(8)	-15.5(7)	14.0(6)	-12.2(6)

Table S26. Bond Lengths for **4**.

Atom	Atom	Length/Å	Atom	Atom	Length/Å
F1	C7	1.319(3)	C4	C8	1.501(2)
F2	C7	1.324(3)	C5	C6	1.377(3)
F3	C7	1.318(3)	C9	C10	1.502(2)
O1	C8	1.220(2)	C10	C11	1.543(2)
O2	C9	1.210(2)	C10	C13	1.552(2)
O3	C12	1.195(2)	C11	C12	1.512(2)
N1	N2	1.3853(18)	C11	C16	1.557(3)
N1	C8	1.342(2)	C13	C14	1.497(3)
N2	C9	1.381(2)	C13	C18	1.528(3)
N2	C12	1.398(2)	C14	C15	1.321(3)
C1	C2	1.368(3)	C15	C16	1.496(3)
C1	C6	1.368(3)	C16	C17	1.532(3)
C1	C7	1.496(2)	C17	C18	1.507(3)
C2	C3	1.383(2)	C17	C19	1.500(3)
C3	C4	1.373(2)	C18	C19	1.498(3)
C4	C5	1.384(3)			

Table S27. Bond Angles for **4**.

Atom	Atom	Atom	Angle/°	Atom	Atom	Atom	Angle/°
C8	N1	N2	119.11(14)	N2	C9	C10	107.66(14)
N1	N2	C12	122.94(14)	C9	C10	C11	105.24(13)
C9	N2	N1	122.08(14)	C9	C10	C13	111.67(14)
C9	N2	C12	114.83(13)	C11	C10	C13	110.07(13)
C2	C1	C6	119.97(17)	C10	C11	C16	108.81(14)
C2	C1	C7	120.74(18)	C12	C11	C10	105.46(13)
C6	C1	C7	119.25(18)	C12	C11	C16	113.04(15)
C1	C2	C3	120.11(18)	O3	C12	N2	123.75(15)
C4	C3	C2	120.57(17)	O3	C12	C11	129.58(16)
C3	C4	C5	118.65(16)	N2	C12	C11	106.66(14)
C3	C4	C8	124.04(16)	C14	C13	C10	106.30(13)
C5	C4	C8	117.30(16)	C14	C13	C18	111.09(15)
C6	C5	C4	120.68(19)	C18	C13	C10	103.68(15)
C1	C6	C5	120.01(19)	C15	C14	C13	114.75(17)
F1	C7	F2	104.1(2)	C14	C15	C16	114.94(17)
F1	C7	C1	113.30(19)	C15	C16	C11	107.30(14)
F2	C7	C1	112.82(18)	C15	C16	C17	110.71(17)
F3	C7	F1	108.5(2)	C17	C16	C11	103.68(15)
F3	C7	F2	105.2(2)	C18	C17	C16	110.28(15)
F3	C7	C1	112.3(2)	C19	C17	C16	122.22(19)
O1	C8	N1	122.77(16)	C19	C17	C18	59.75(14)
O1	C8	C4	121.17(16)	C17	C18	C13	110.70(15)

Atom	Atom	Atom	Angle/°	Atom	Atom	Atom	Angle/°
N1	C8	C4	116.03(14)	C19	C18	C13	121.62(19)
O2	C9	N2	123.87(15)	C19	C18	C17	59.90(15)
O2	C9	C10	128.47(15)	C18	C19	C17	60.36(14)

Table S28. Hydrogen Bonds for **4**.

D	H	A	d(D-H)/Å	d(H-A)/Å	d(D-A)/Å	D-H-A/°
N1	H1	O4	0.86(2)	1.94(2)	2.767(2)	163(2)
C2	H2	F1[1]	0.93	2.58	3.364(3)	142.4
C3	H3	O4	0.93	2.60	3.495(2)	162.3
C10	H10	O2[2]	0.98	2.49	3.326(2)	142.7
C11	H11	O3[3]	0.98	2.56	3.368(2)	140.2
C13	H13	F2[4]	0.98	2.65	3.302(2)	124.4
C15	H15	O2[5]	0.93	2.54	3.284(2)	137.5
O4	H4A	O1[6]	0.851(17)	1.937(18)	2.7853(19)	174(3)
O4	H4B	O2[5]	0.859(17)	2.042(19)	2.886(2)	167(3)

[1]1/2-X,+Y,1-Z; [2]3/2-X,3/2-Y,1/2-Z; [3]3/2-X,1/2-Y,1/2-Z; [4]+X,3/2-Y,-1/2+Z; [5]1-X,-1/2+Y,1/2-Z; [6]-1/2+X,1-Y,+Z

Table S29. Torsion Angles for **4**.

A	B	C	D	Angle/°	A	B	C	D	Angle/°
O2	C9	C10	C11	-176.36(17)	C9	C10	C11	C16	119.00(14)
O2	C9	C10	C13	-57.0(2)	C9	C10	C13	C14	-60.33(18)
N1	N2	C9	O2	0.7(3)	C9	C10	C13	C18	-177.52(14)
N1	N2	C9	C10	-179.36(14)	C10	C11	C12	O3	-179.9(2)
N1	N2	C12	O3	-2.0(3)	C10	C11	C12	N2	0.57(18)
N1	N2	C12	C11	177.61(14)	C10	C11	C16	C15	-54.04(18)
N2	N1	C8	O1	-1.7(3)	C10	C11	C16	C17	63.15(18)
N2	N1	C8	C4	176.56(15)	C10	C13	C14	C15	-58.6(2)
N2	C9	C10	C11	3.68(17)	C10	C13	C18	C17	63.0(2)
N2	C9	C10	C13	123.09(15)	C10	C13	C18	C19	129.6(2)
C1	C2	C3	C4	0.7(3)	C11	C10	C13	C14	56.18(18)
C2	C1	C6	C5	-1.3(4)	C11	C10	C13	C18	-61.01(17)
C2	C1	C7	F1	16.7(3)	C11	C16	C17	C18	-62.6(2)
C2	C1	C7	F2	134.7(2)	C11	C16	C17	C19	-128.9(2)
C2	C1	C7	F3	-106.7(3)	C12	N2	C9	O2	176.41(16)
C2	C3	C4	C5	-1.4(3)	C12	N2	C9	C10	-3.63(19)
C2	C3	C4	C8	179.95(19)	C12	C11	C16	C15	62.77(19)
C3	C4	C5	C6	0.8(4)	C12	C11	C16	C17	179.96(15)
C3	C4	C8	O1	-171.9(2)	C13	C10	C11	C12	-123.01(14)
C3	C4	C8	N1	9.8(3)	C13	C10	C11	C16	-1.46(18)
C4	C5	C6	C1	0.6(5)	C13	C14	C15	C16	0.1(2)
C5	C4	C8	O1	9.4(3)	C13	C18	C19	C17	-97.1(2)
C5	C4	C8	N1	-168.9(2)	C14	C13	C18	C17	-50.8(2)
C6	C1	C2	C3	0.6(3)	C14	C13	C18	C19	15.8(3)
C6	C1	C7	F1	-165.9(2)	C14	C15	C16	C11	58.4(2)
C6	C1	C7	F2	-47.9(3)	C14	C15	C16	C17	-54.1(2)
C6	C1	C7	F3	70.7(3)	C15	C16	C17	C18	52.2(2)

A	B	C	D	Angle/°	A	B	C	D	Angle/°
C7	C1	C2	C3	178.0(2)	C15	C16	C17	C19	-14.1(3)
C7	C1	C6	C5	-178.7(3)	C16	C11	C12	O3	61.3(3)
C8	N1	N2	C9	-78.9(2)	C16	C11	C12	N2	-118.19(16)
C8	N1	N2	C12	105.7(2)	C16	C17	C18	C13	-0.9(2)
C8	C4	C5	C6	179.5(3)	C16	C17	C18	C19	-116.3(2)
C9	N2	C12	O3	-177.66(18)	C16	C17	C19	C18	96.1(2)
C9	N2	C12	C11	1.9(2)	C18	C13	C14	C15	53.6(2)
C9	C10	C11	C12	-2.55(17)	C19	C17	C18	C13	115.4(2)

Table S30. Hydrogen Atom Coordinates (Å×10^4) and Isotropic Displacement Parameters (Å^2×10^3) for **4**.

Atom	x	y	z	U(eq)
H1	5115(18)	5210(20)	3296(8)	50(6)
H2	3543.64	5982.67	4665.68	63
H3	4346.79	5471.08	3947.91	59
H5	6907.05	7272.69	4495.41	88
H6	6089.56	7812.82	5203.2	93
H10	7621.76	5951.94	2085.95	46
H11	7641.54	3886.35	2228.28	50
H13	6248.85	6813.78	1509.35	53
H14	4623.74	5816.31	1581.76	59
H15	4618.91	3652.55	1746.9	62
H16	6233.55	2589.38	1833.06	60
H17	7536.22	3319.17	1282.24	69
H18	7552.84	5553.78	1117.71	66
H19A	6691.95	4139.18	519.08	80
H19B	5606.87	4300.29	792.36	80
H4A	3278(18)	4320(30)	3259(10)	84
H4B	3890(20)	3850(20)	2890(10)	84

Table S31. Interaction energy calculation results for **1** with CE-1p model

Count	Distance	Description	Coulomb	Dispersion	Exchange	Polarization	Repulsion	Total
2	7	1A : 1A1/2+x,1/2-y,1-z	-29.2	-11.4	-30.8	-5.5	53.8	-33.9
1	4.3	1A : 1A-x,-y,1-z	-9.8	-29	-24.7	-1.4	43.2	-31.1
2	5.34	1A : 1A 3/2-x,-1/2+y,z	-13.4	-19.3	-21.7	-2.2	39	-25.8
2	8	1A : 1A-x,-1/2+y,1/2-z	-2.5	-7.9	-4	-0.3	7.9	-8.6
2	8.06	1A : 1A1/2+x,y,1/2-z	-2.4	-5.8	-3.6	-0.3	6.5	-6.9
1	8.8	1A : 1A-x,-1-y,1-z	4	-5.9	-2.5	-0.6	4.8	-1.2

Table S32. Interaction energy calculation results for **2** with CE-1p model

Count	Distance	Description	Coulomb	Dispersion	Exchange	Polarization	Repulsion	Total
2	4.89	1A : 1Ax,-1+y,z	-47	-41.7	-66.6	-8.3	120.5	-68
2	5.15	1A : 1A-1+x,y,z	0.5	-42.9	-25.1	-1.6	45.3	-33.4
2	7.1	1A : 1A-1+x,-1+y,z	-15.2	-15.9	-18.5	-2.3	30.9	-27.3
4	16.11	1A : 1A-x,-1/2+y,1/2-z	-1.7	-4	-1.9	-0.2	3.5	-5.0

4	18.41	1A : 1A-3-x,1/2+y,3/2-z	0.6	-3	-0.5	-0.1	1.3	-1.9

Table S33. Interaction energy calculation results for **3** with CE-1p model

Count	Distance	Description	Coulomb	Dispersion	Exchange	Polarization	Repulsion	Total
1	4.8	1A : 1A 1-x,1-y,1-z	-28.2	-48.7	-45.6	-6.1	78.7	-65.9
1	6.6	1A : 1A-x,1-y,1-z	-20.6	-27.5	-31.7	-7.4	55.6	-42.3
1	6.2	1A : 1A1-x,-y,1-z	-13.4	-25.2	-20.8	-2.3	33.5	-35.2
1	5.5	1A : 1A-x,-y,1-z	-2.0	-33.0	-24.2	-3.4	44.5	-27.2
2	6.6	1A : 1A-1+x,y,z	-3.0	-17.4	-11.4	-1.5	18.9	-18.2
2	16.3	1A : 1A-1+x,y,-1+z	-2.6	-4.8	-3.0	-0.3	5.4	-6.5
1	14.8	1A : 1A1-x,1-y,2-z	-0.3	-6.7	-3.6	-0.2	6.6	-5.6

Table S34. Interaction energy calculation results for **4** with CE-1p model

Count	Distance	Description	Coulomb	Dispersion	Exchange	Polarization	Repulsion	Total
1	6.6	1A : 1A1/2-x,3/2-y,2-z	-21.8	-22.4	-20.2	-5.2	35.6	-40.9
2	6.2	1A : 1A 1/2-x,-1/2+y,1/2-z	-10.8	-28.9	-24.2	-3.0	41.8	-33.7
1	8.2	1A : 1A1/2-x,5/2-y,2-z	-18.5	-16.4	-18.3	-3.5	32.2	-30.7
1	11.3	1A : 1A 1-x,1-y,1-z	-2.5	-28.5	-19.4	-1.0	35.3	-23.8
2	6.6	1A : 1Ax,1-y,-1/2+z	-1.6	-12.7	-2.7	-1.5	4.0	-15.1
2	14.0	1A:1A-1/2+x,3/2-y,-1/2+z	-2.8	-9.2	-5.8	-0.3	10.4	-10.0
1	14.4	1A : 1A1-x,y,-1/2-z	-4.9	-6.2	-6.5	-0.5	11.7	-8.8
2	16.1	1A : 1A-1/2+x,-1/2+y,-1+z	-2.1	-5.0	-2.3	-0.3	4.2	-6.4
1	11.3	1A : 1A 1-x,y,1/2-z	2.8	-8.0	-3.8	-0.7	5.8	-5.0
